# Vanishing social classes? Facts and figures of the Italian labour market

**DOI:** 10.1007/s00191-022-00793-4

**Published:** 2022-11-17

**Authors:** A. Cetrulo, A. Sbardella, M. E. Virgillito

**Affiliations:** 1 Institute of Economics, Scuola Superiore Sant’Anna, Piazza Martiri della Libertà, 33, 56127 Pisa, Italy; 2 CNAM CEET, Paris, France; 3 Enrico Fermi Research Center, Rome, Italy; 4 Università Cattolica del Sacro Cuore, Milan, Italy

**Keywords:** Inequality, Wages, Occupations, E24, J31, J50

## Abstract

This paper analyses medium-term labour market trends from 1983 to 2018 in Italy relying on the “Rilevazione dei contratti di lavoro” from INPS archive which provides information on average salaries by professional category, age, gender, and geographical origin. Within an overall pattern of exacerbated wage inequalities, documented by means of different indicators, the empirical analysis highlights how the *within*-component of the wage variation prevails in the gender, age and geographical dimensions. By contrast, the *between-*component in terms of professional categories (trainees, blue-collar jobs, white-collar jobs, middle managers, executives) is the only between-variation attribute to prevail, corroborating the role played by a reduced class schema, excluding capitalists and the self-employed, in explaining wage inequality. Regression-based inequality estimations confirm the role played by managerial remuneration, the contradictory located class, in driving divergent patterns. Stratification of wage losses is recorded to be largely concentrated among blue-collar professional categories, women, youth, and in Southern regions.

## Introduction

From the 1980s onwards both sociology and economics have witnessed a gradual marginalization of the role and centrality of social classes as lens of analysis in understanding, on the one hand, structural transformations in the employment composition and, on the other hand, socio-economic conditions and income evolution (Pugliese 2008). With reference to Italy, with the exception of the pioneering contribution on the Italian occupational structure by Sylos Labini (1974, 2014), social classes have been largely dismissed in recent analyses, while inequality has revamped and gained a great deal of attention in the public and scholarly debate. No doubt, the underlying difficulty to operationalize the notion of social classes might have reinforced its abandonment in social sciences.

Several scholars questioned the empirical validity and theoretical relevance of a class-based approach in explaining the key challenges of contemporaneous society and new inequality trends arguing that social class analysis is not able to account for increasing individualism, lack of political identity and activism (Beck-Gernsheim and Beck 2001). Therefore, the class-based approach should be historically confined to the description of industrial societies now progressively overtaken by a post-industrialism and post-modernism era (Pakulski and Waters 1996; Pakulski 2005). In addition, different theories asserting the *death of classes* have been put forward (Eidlin 2014). Nonetheless, the analysis via social classes, mainly framed in terms of occupational categories, is still crucial to understand the undergoing transformations of society (Wright 1998; Grusky and Weeden 2001; Atkinson 2007). Least but not last, social classes and occupational categories have been shown to be particularly relevant in analyzing the COVID-19 pandemic phase, especially in terms of access to telework (Cetrulo et al. 2022) and, more in general, in studying educational opportunities, healthcare access, and intergenerational transmission of status (Albertini 2013). Furthermore, the interaction between micro-level occupational structures and macro-level class schemes has been recently adopted as interpretative lens in examining the anatomy of Italian occupations (Cetrulo et al. 2020a).

By using administrative data on Italian wages and jobs (INPS Longitudinal Sample – *Rilevazione dei contratti di lavoro*), we decline the notion of social classes via the underlying employment relations they entail, and we focus, among the group of employees, on the different occupational hierarchical ladders, in line with recent research on social classes (Albertini 2013). We look at the Italian labour market in the medium-run (1983–2018), and we intersect three interrelated dimensions: social classes – intended as blue-collar, white-collar, managerial and business executive macro-occupational categories –, their remuneration in terms of wages, and their attributes in terms of industry characterisation, gender, age, type of job contract, and regional distribution.

Our results, within an overall picture of declining real wages, reduced number of working weeks and increasing number of jobs, highlight severe processes of divergences in terms of (i) wage distribution between white-collars and blue-collars versus executives, (ii) top versus bottom decile of the wage distribution, (iii) sectoral dynamics, (iv) gender and age divides. If the gap with respect to the top of the employment distribution tends to increase over time, some patterns of convergence versus the low-end appear, particularly after the 2008 crisis, in terms of decreasing median–bottom wage gaps.

In terms of periodization, while some patterns of divergence exploded with the 2008 crisis – in particular the ‘proletarization’ of middle-wage occupations –, others, such as declining wages and jobs for young versus elderly workers, definitely pre-date the 2008 crisis. Indeed, the gradual process of market flexibilization started at the beginning of the 1990s has resulted in a strong increase of part-time and short-term contracts, particularly among women in Southern Italy who register the lowest wages across all worker categories.

Within a general trend of exacerbated wage inequalities,^1^ our empirical analysis highlights how the within-component of the wage variation prevails in the gender, age and geographical dimensions. By contrast, the between-component in terms of professional categories (trainees, blue-collar jobs, white-collar jobs, middle managers, executives) is the only prevailing between-variation attribute, thereby providing evidence on the fact that the professional dimension still represents the greatest source of wage inequality. Regression-based inequality estimations confirm the role played by social classes. Moreover, the stratification of wage losses is recorded to be largely concentrated among blue-collar professional categories, women, youth, and in the Southern regions.

Our analysis is based on employee wage data and therefore does not take into account (i) other sources of income that contribute in explaining income inequality, (ii) employers’ jobs, (iii) autonomous jobs. Although this might represent a limitation in understanding the overall dynamics of social classes, it is worthy to restrict the focus to wages only, since our interest lies in identifying eventual patterns of convergence/divergence in wages resulting from different positionings along the hierarchy of employment relationship. In addition, considering the presence of managerial positions in our dataset and the increasing decision-making role exerted by the latter in business organizations, managers and executives represent the hierarchical ladder most akin to employers.

Finally and contrary to the purported pattern of sheer polarization put forward by mainstream labour economics (Acemoglu and Autor 2011), we do not find descriptive evidence of market-based competitive forces (primarily technology and education) driving wage inequality – which should be reflected in U-shaped wage and employment changes along wage percentiles: changes in wages by wage distribution do not follow changes in occupations by wage distribution. By contrast, we detect a remarked tendency towards wage compression, resulting in a generalised negative wage growth, except for the very top percentile. This result leaves room for taking into account other institutionally and structurally based determinants of wage inequality, far from market-based forces.

The remainder of the paper is organised as follows. Section 2 provides a synthetic overview on the debate on social classes and labour process empirical analysis. Section 3 describes the employed dataset, while Section 4 discusses facts and figures of the Italian labour market. Determinants of wage inequality are explored in Section 5. Section 6 presents our conclusions.

## Social classes inside productive units: labour process and managerial functions

In the following we first lay out alternative theories of social classes in Subsection 2.1; we then present the empirics of social classes in Subsection 2.2; finally, we delve into the role of management as a functional category for a contemporary class schema in Subsection 2.3.

### Alternative theories of social classes

The analyses of class formation and identities have experienced alternate successes, with phases of rise and fall. Two views are largely present in the debate. The *end of class approach* according to which social classes have progressively become weak theoretical categories, since they are considered inadequate to describe a post-industrialism phase characterised by rising individualism and increasing importance of identities over material attributes. Alternatively, *the defeat of class approach* postulates that social classes, rather than being irrelevant, are in retreat as a result of the weakening of political organizations, mainly unions and labour movements, providing them adequate space for organizing (Eidlin 2014, see Table 1 for a synthetic description).

**Table 1:** Alternative theories of social classes. Own elaboration from Eidlin (2014)

Phase	Theory	Mechanism of class formation	Approach	Actors
XIX century	Marxist view	Means of production	Materialist-Relational	Working class
Pre-WWII	Weber view	Positional attributes in the market	Symbolic-Relational	High-prestige vs low-prestige individuals
Post-WWII	Lipset view	Individual attributes	Gradational	Low-income vs high-income owners
Post-industrialism	End of class view	Control over knowledge	Post-materialism	Knowledge workers
	Wright view	Contradictory class location	Materialist-Relational	New middle class
	New Social Movements view	Individual identities	Symbolic-Relational	The people
	Bourdieu view	Field	Symbolic-Relational	The dominators vs the dominated

However, moving beyond the different and often contested views on social classes, the current phase of capitalism – unable to keep sustaining social mobility and characterised by deep and stratified asymmetries – calls, yet again, for a social class approach to the study of wage inequality. In fact, the theory of social classes offers different possible explanations for the origin of inequality and the functioning of capitalism when compared to mainstream approaches to labour markets based solely on technology and education (Papagiannaki et al. 2020). More recently, scholars have been deeply questioning the adoption of a single methodology to effectively grasp the key determinants and characteristics of growing inequalities. The need of a “multidimensional approach” is evoked for distinguishing several “spaces” of disparities shaped by unbalanced endowment of economic, cultural, social and physical assets (Grusky 2018). This controversial debate intersects with increasing research over job polarization trends (Autor et al. 2006), middle class shrinking (Vaughan-Whitehead 2016) or professional and managerial jobs expansion (Ikeler and Limonic 2018; Oesch 2022), growing inequality at the top (Alvaredo et al. 2013), intersectionality and social stratification within advanced economies (McCall 2002).

The first dimension to consider when focusing on social classes concerns its definition in “relational” or “gradational” terms (Wright 1979; Eidlin 2014). Both Marx and Weber, two of the most prominent theorists of social classes, share a relational view to social classes, which are thought to build their identity in relation/conflict/opposition to the other classes. In both theories, the relational approach makes explicit the positional role of social classes *among* them, because of the specific relations that exist within the structure of interests that defines a given economic system – essentially shaped by productive and reproductive forces in Marx, and markets in Weber. However, the two theories have different interpretations of the process of identity formation. In Marx identities are rooted in economic materialism and commonality in workplace activities (*class in itself*) which should eventually spur into the *class for itself*, the proletariat appropriating the means of productions. While, in Weber they are considered to be based on market contexts and positional individual attributes, whereby the struggle is in terms of prestige, and cultural and symbolic elements supersede materialistic motives of class identity. Weberian and Neo-Weberian approaches see market exchange relations as determinants of individual life chances, depending on the personal availability of assets and skills (Breen 2005). According to Weber, classes are not “communities” but essentially sets of individuals sharing common economic interests in owning goods and services that are exchanged in the (commodity and labour) markets and then determine individual life chances (Weber 2018).

After WWII, the approach to social classes, also as a consequence of the advent of mass society and culture, largely turned into gradational (Lipset 1960). In the gradational approach, the position of each group depends on the degree or intensity of a specific characterising attribute, such as the level of income, skills, or the endowment of authority. The most prominent studies adopting a gradational perspective make use of occupational prestige scales (Treiman 2013) or socioeconomic scales (Duncan and Reiss 1961; Hauser and Warren 1997) to highlight differences across occupations. Today, a gradational approach that looks at income inequality trends without engaging any theoretical discussion over the origin of social classes is widespread, also given the increasing attention devoted to inequality in the mainstream debate, that, as mentioned, is largely focusing on skills (Katz and Murphy 1992) and technology (Autor 2015b).

With the advent in the Western world of the so-called post-industrial society, the notion of class has been profoundly challenged. On the one hand, place- and field-based approaches a’la Bourdieu, still rooted into the relational view, questioned the materialistic interpretation of class identity which was viewed as too deterministic and moved towards the study of the spatiality of power, practices and systems of dominance reproduction. On the other hand, the New Social Movements theory (Eidlin 2014), largely based on shared identities and views (race, gender and environmentalism) across classes, put forward the notion of “The People”, rather than the working class, as actor of change. However, as will be discussed in the following, social classes remain relevant theoretical and empirical categories to study the new world of work, progressively far from factories and more service based, and from the 1980s increasingly subject to non-standard forms of employment relationships.

### The empirics of social classes

Goldthorpe’s class schema can be considered as one of the most accurate and diffused operationalization of the Weberian approach to social classes (Goldthorpe et al. 1980; Erikson et al. 1992). Despite its different formulations, the main criteria behind this class schema are the positions that individuals hold in the labour market (i.e., occupation and income level) and in the organization of work (distinguishing for degree of authority, level of skills, nature of activity, etc.). Accordingly, workers are first divided into employees, employers and self-employed. The employees’ group is further decomposed according to i) the type of employment relation regulation, whether corresponding to service, intermediate or labour contract; and ii) the degree of the authority exercised, the skill level, the intensity of supervision, the relevance of manual/non manual activities and routinariety (Erikson et al. 1992). This class schema has been used in the literature with different degrees of aggregation, going from four broad categories (manual, service, intermediate class and petty bourgeoisie) to more complex seven or eleven groups class schema, where particular attention is devoted to job content and skills.^2^

A similar effort in operationalising a class analysis however with a Marxian lens was made by Wright (2000). His class schema resorts to two main concepts: authority in the production process and endowment of skills and expertise. More precisely, with his 12-class locations map, Wright distinguishes individuals based on i) their relations to the means of production (owners or employees); ii) authority (managers, supervisors, non management); iii) skills (experts, skilled, non-skilled). He further distinguishes owners of the means of production in capitalists, small employers and petty bourgeoisie according to their number of employees (Wright 2000) [p.22]. Despite their similarities, Goldthorpe’s and Wright’s class maps rest on different theoretical foundations. According to the Marxist perspective embraced by Wright, the space where social classes get defined is the sphere of production, rather than the market, because the former is able to capture the underlying exploitation mechanism inherent in the capitalist system. While the two broad categories of working class/proletariat (namely those obliged to sell their labour in exchange for a wage to ensure survival) and bourgeoisie/capitalists (namely the owners of the means of production that get profits) could be easily placed at two opposite poles, the on-going transformations in labour markets make it more difficult to adopt a clear-cut class scheme. Therefore, social relations of production can be further characterised as social relations of control over i) money capital (i.e. investment and accumulation); ii) physical capital (i.e. means of production); iii) the labour process (Wright 1979).

An attempt to reconcile class analysis and heterogeneity within a class framework, especially for what concerns lifestyle and cultural differences (Bourdieu 1987), is the one proposed by neo-Durkheimian scholars (Grusky and Weeden 2001). According to these authors, the alleged weakness of a class-based approach resides essentially in the level of aggregation: macro classes schemes are not able to describe the effective mechanisms (allocation, social conditioning, etc.) through which social stratification takes place and risk to remain only abstract categories (Weeden and Grusky 2005). Nevertheless, the labour market is still conceived as the main locus where social classes arise, even if a more disaggregated level of analysis – namely occupations – should be preferred, as occupations can be viewed as “micro-classes embedded in the very fabric of society” and therefore can be “meaningful not merely to sociologists but to the lay public as well” (Grusky and Weeden 2001) [pp. 203–204]. The increasing number of occupations’ closure mechanisms, such as the adoption of licenses and credentials, is usually interpreted as further evidence in favor of such units of analysis (Weeden 2002).

Interestingly, if on the one hand these studies seem to downsize the explanatory power of macro-categories, on the other hand, they see the disaggregation approach as complementary and not alternative for a comprehensive social-classes analysis, that remains essentially based on a relational rather than gradational perspective.

### Contradictory class locations: managers as a class

With the advent of the third industrial revolution, modern capitalism increasingly became more centered on knowledge accumulation. In addition, control over knowledge and the whole labour process, albeit already clear in the Marxian analysis, increased its importance and progressively got separated from ownership of money and physical capital. The rise of managerial capitalism and the consequent emergence of modern firms (Chandler 1993), where managerial functions gradually gained a central role in creating the hierarchical structure of the firm as well as technical and job assignments, deeply affected workers’ bargaining power (Marglin 1974; Braverman 1974).

To understand the role of managers in the workplace as controllers and regulators of the labour process, Wright proposes the emergence of “contradictory class locations”, whereby the notion of contradiction refers to the rise of antagonistic interests shaped by structural underlying tensions inside the “new middle class”. In order to tame potential tensions within their socio-technical functions, the problem of “controlling the controllers” arises. Repressive mechanisms of surveillance, however, cannot be easily enforced against managers and are instead replaced by specific inducements and rewarding schemes (Wright 1979) [pp. 89–91] to create obedience and coalescence, which are considered essential to run the firm (Dosi et al. 2021c). The growing high wage differentials between managers and the rest of the workforce, even with similar educational attainments, should thus be interpreted as “an income privilege component in their wage (...) which lowers their rate of exploitation and sets them off from the working class at the level of exchange relations” (Wright 1979) [p. 227]. Therefore, the more complex the hierarchical structure of the managerial layer, the bigger the wage gap and the wider the composition of the wage setting will be along the hierarchy, as different levels of remunerations are supposed to increase the legitimacy and institutionalization of the authority structure (i.e., granting stock options to CEOs).

The role of managers and the presence of competing interests within productive organizations is also stressed by corporate governance theories that, refusing a reductionist interpretation based on the efficiency contract framework, stress how corporate governance reflects an unbalanced distribution of political and economic resources among investors, top managers and workers (Jung 2016). Accordingly, when important decisions that alter the interests and resources of the involved actors (i.e. workforce downsizing) are announced, unexpected outcomes can be foreshadowed. In particular, the role of top managers seems to be crucial, as they share interests both with investors (maximizing profits and therefore their compensation) and workers (ensuring job stability but mostly preserving managerial autonomy).

Accounting for different authority endowments is crucial to tackle the contradictory nature of class locations, such as that of managers, who are granted the “privilege” and power to self-appropriate higher portions of the surplus, despite the exploitative employment relation to which they are subject to (Wright 2000). In that respect, they constitute an occupational layer worth to be studied, as they occupy an autonomous and distinct location within the middle class.

## Methodology and data

Moving forward, in Subsection 3.1 we discuss our empirical social class-based framework, while in Subsection 3.2 we present a description of the dataset employed. 

### Methodology

In this paper, taking a relational approach, we focus on social relations of production, looking in particular at the notion of control over the labour process and distinguishing for the authority and knowledge dimension. Our social class scheme – based on the five main categories of trainees, blue-collars, white-collars, middle managers and executives – allows us to identify salient traits of a class-based analysis, given the significant heterogeneity of the macro-categories in the domains of interest. The dichotomy between blue-collars and white-collars is well established in the socio-economic literature, both in terms of the content of the job activity (Oesch 2006), the intensity of the skills required, the job mobility and the career opportunities (Gallie 1996), and in terms of the potential impact of technological change on their activity (Smith 1991). Given their different degrees of authority and positional power, middle managers and executives represent closely located but still different strata within the middle class. Indeed, white-collars, middle managers and executives can be seen as lower, core and upper middle class (Atkinson and Brandolini 2013), while blue-collars can be easily juxtaposed with the notion of working class. Lastly, trainees represent an interesting category to be distinguished from blue-collars, given their involvement in specific programs directly supplied by firms, aimed at increasing their professional skills and easing their entrance in the labour market (D’Agostino and Vaccaro 2021).

Our social class framework refers exclusively to employees, as we do not have any information on capitalists and self-employed workers. This choice – which is also linked to data availability – has the disadvantage of preventing the adoption of a broader classification of social classes, that would include capitalists and the petty bourgeoisie. Moreover, given the absence of data on assets’ property rights, the role played by rents is beyond the scope of the analysis, despite its relevance with respect to wealth accumulation and therefore in shaping life conditions (Sørensen 2000, 2018). As we have documented in the previous sections, whether occupations truthfully reflect social classes is still open to debate. However, despite these potential drawbacks, we believe our choice and framework are valid for three main reasons.

First, the years under study represent a new phase of re-organization of labour markets and their institutions, in Italy and abroad, as testified by the large number of legislative acts directly targeting labour organisations and institutions with the explicit aim of increasing flexibility and weakening workers’ bargaining power (Howell 2021), namely the neo-liberal phase. A reduced class schema that exclusively considers those who sell their labour power, who were purposely affected by these reforms, allows us to account for the asymmetric effects of wage compression and labour power erosion across class relations.

Second, given that the progressive dispossession of workers’ degree of control and autonomy over the labour process is one fundamental trait of capitalist economies (Braverman 1974; Marglin 1974), studying how the wage distribution changes across the hierarchical dimension emerging from the social division of labour is complementary to the study of different sources of heterogeneity related not only to wages, but also to the inner characteristics of the Italian occupational structure (Cetrulo et al. 2020a). Furthermore, distinguishing among white-collars, middle managers and executives enables us to better qualify the internal composition of the middle-class, whose pattern of growth is still an object of discussion (Simonazzi and Barbieri 2016), and whose contradictory location can be interpreted as part of capitalism evolution (Harvey 2007; Ikeler and Limonic 2018; Lévy and Duménil 2018).

Third, occupational analysis remains the most widespread approach to study social classes and social stratification, with occupational layers representing predictors of wage inequality (Avent-Holt et al. 2020). Although occupations are far from being a subjective dimension of class identity (class for itself), they do represent an objective, materialistic dimension (class in itself). Therefore, we deem appropriate and of interest to study their dynamics.

### Dataset description

We rely on the Italian Institute of Social Security Longitudinal Sample – *Rilevazione dei contratti di lavoro*, a high-quality micro-aggregated level data based on administrative records. As its name suggests, the dataset has a longitudinal structure and is based on a large representative sample of employees in the private sector – with the exception of agricultural and domestic jobs – from 1982 to 2018. Therefore, it does not include information on public employees or, as mentioned above, any type of self-employed jobs.

For each year the *Rilevazione dei contratti di lavoro* open archive^3^ contains information on the number of jobs, yearly or weekly gross salaries and weeks of work (especially relevant for part-time and intermittent jobs) as reported by private-sector employers, together with a number of socio-professional characteristics of the job, such as gender, age, typology of employment, region and economic sector of activity.

While the micro-level version of this dataset reports individual data, our analysis is based on the publicly available (upon request) data, which contains information averaged over different segments of the workforce. Hence, we are not able to follow individual workers’ employment histories, but we observe more aggregated characteristics of the labour force.

Each observation of our dataset is characterised by five of the aforementioned socio-professional variables – region (20 Italian regions), geographical area (North-West, North-East, South, Center, Islands), gender, age cohort (under 30, 30–50, over 50 years old), and occupational status (trainees, blue-collars, white-collars, middle managers, executives) – that identify specific segments of the labour force. In each year, the theoretical maximum number of segments given by all the combinations of the socio-professional characteristics is 600 (20 regions, 5 occupations, 3 age groups, 2 genders). However, when excluding missing values and considering the fact that the managerial occupational category was introduced only in 1996, we obtain a total of 17,371 observations with the actual maximum number of cells being 559 in 2003 and the minimum 344 in 1995. For all those combinations, in each year, we perfom weighted averages of the taxable amount of each worker’s wage, the weeks actually remunerated in the year, the labour market entry age, the percentage of part-time (since 1985) and permanent (since 1998) jobs, the percentage of jobs in 1-digit ATECO 2007 economic sectors (i.e., Mining and Quarrying; Manufacturing; Metallurgy, Chemical and Pharmaceutical industries; Energy; Water Supply; Sewerage and Machinery; Construction, Wholesale and Retail Trade; Transport; Information and Communication together with Financial and Insurance Activities; Professional Services; Education and Human Health Activities; Other Services). For instance, a single cell of our dataset may detail for under 30 years old, white-collar women working in Sicily in 2018 the number of jobs on which the averages of the gross real salary were received and the weeks of work were performed, together with information on sectoral and type of employment shares.

To construct our sample, we focus primarily on the occupational structure as interpretative lens for the undergoing transformations in the Italian labour market, and on the interaction of the macro-occupations with the geographical area, type of employment, gender, age cohort and sectors in determining the distribution of wages, that are expressed in real terms in constant 2016 euros.

Given that we do not look at the individual dimension, to maximise the informative content of the data, we take into account the possibility of secondary or tertiary jobs and compute the averages using as weights all the employment relationships in each segment of the labour force, rather than considering only primary jobs. Indeed, following the increasing weakening of the labour market regulation and the steady rise of non-standard jobs, accounting for primary jobs only and not for total jobs might lead to an under-estimation of the real number of employment relations activated for a given category of worker in a given year.

**Fig. 1:**
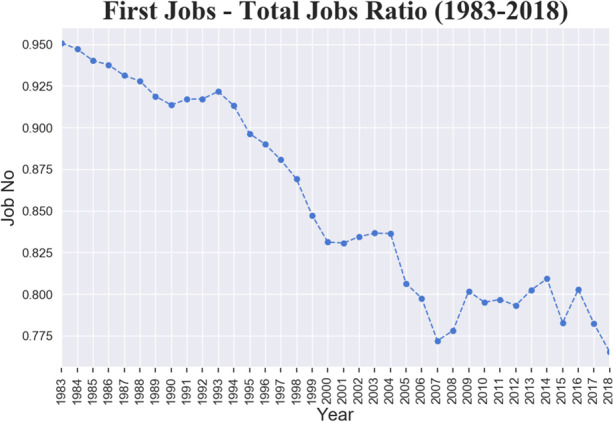
Ratio between number of first jobs and total number of jobs. Italy, 1983–2018

To motivate our choice, Fig. 1 presents the ratio between the number of first over total number of jobs (that may include secondary or tertiary jobs). The trend is clearly decreasing since the 1980s, ranging from 95% in 1982 to 77% in 2018. However, the pace of the fall changes distinctively in three different periods, reflecting the process of job-fragmentation and the appearance of open-ended contracts: up to 1995 (5% drop), from 1995 to 2008 (13% drop), after 2008 (constant trend). Considering that secondary and tertiary jobs are concentrated among low paid occupations, their exclusion would upward bias the analysis.^4^

**Fig. 2:**
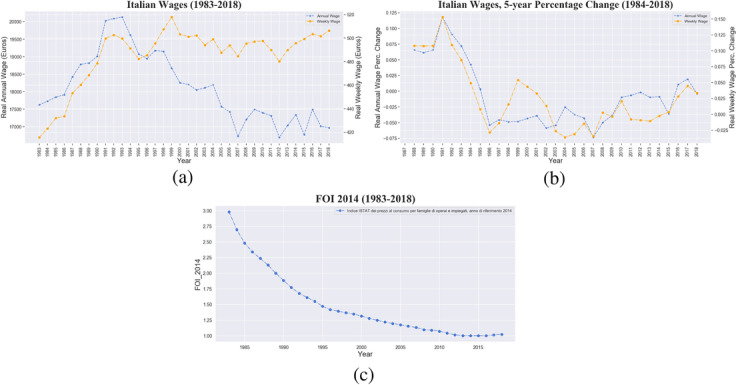
Italy, 1983–2018, wage compression trend. **a** Real average wage (annual in blue, weekly in orange). **b** Real average wage 5-year percentage change (annual in blue, weekly in orange). **c** FOI, price index used to compute real wages

Finally and as mentioned above, in our analysis we focus exclusively on yearly earnings. Despite the crucial role played by other sources of income, such as wealth (Acciari et al. 2020), we deem an analysis on wages extremely informative as they represent the principal source of disposable income for the majority of workers (Quintano et al. 2009), a major trigger of unequal distribution dynamics (Galbraith and Kum 2003), and ultimately reflect the actual remuneration of productive labour in the market. Concerning the unit of scale, weekly and yearly wages can strongly differ as shown in Fig. 2, since the latter are affected by both the level of hourly wages and the total amount of working weeks in a year. However, yearly figures are a more comprehensive measure inasmuch they incorporate the effect of potential wage reduction due to intermittent working activity, e.g. characterised by employment discontinuity or underemployment (as in the case of involuntary part-time or casual jobs).

Table 2 shows descriptive statistics on the number of jobs and the average wage earned for each sub-group belonging to the different population groups described above, summarizing the increasing participation of women into the labour market (8,221 million in 2018 with respect to 2,261 million in 1983); the reduction in youth employment from 2003, as due to both longer educational careers, first-entry job regulated by atypical contracts, and increasing inactivity rates; the leading role of Northern regions that show both higher wages and a larger workforce; and the sharp fall of blue-collars’ average wages (from 13 thousand euro in 1983 to 10 thousand euro in 2018) compared to the substantial increase in wages of middle managers and executives.^5^

## Facts and figures of the Italian labour market

Since the 1980s inequality in both income and wage distributions has been growing at a worrying pace (Franzini and Raitano 2019). The last three decades were marked by major crises (Brandolini et al. 2018), namely the currency crisis in 1992 and the double-dip recession, first with the explosion of the Great Recession in 2008 and then followed by the national debt crisis and the austerity phase. At the same time, profound changes have taken place in the labour market in terms of deregulation of job contracts (Piasna and Myant 2017), deterioration of social dialogue and weakening of industrial relations at the national and European level (Leonardi and Pedersini 2018; Baccaro and Howell 2011). In line with these processes, the Italian labour market underwent a gradual precarisation, in compliance with the European Employment Strategy launched by the European Council in 1994 and sanctioned in the well known OECD (1994) Jobs Study.

Exploiting the longitudinal dimension of our database, we start by identifying and empirically documenting a series of long-run dominant trends that we deem appropriate to characterise the dynamics of the labour market in Italy over the period of observation: wage compression, servitization, job flexibilization and fragmentation, ageing labour force, feminization, geographical divergence, exploding wage inequality.

Figure 2 shows the time evolution of real wages, namely nominal wages deflated over time by the consumer price index FOI (*Indice nazionale dei prezzi al consumo per famiglie di operai e impiegati*). While consumer inflation shown in the bottom part of the panel monotonically shrinks, in the dynamics of yearly and weekly real wages (in blue and orange respectively), three different phases can be detected:

- an increasing trend in the decade 1983–1993;
- an overall declining trend since 1993;
- a strong decoupling between yearly and weekly wages after 1998.

**Table 2:** Yearly average real wages (gross), number of jobs and number of first jobs by workforce sub-group

Categories	Subgroups	Labour market variables	1983	1993	2003	2013	2018
Total		*Average Real Wage*	17,626	20,129	18,111	17,040	16,971
		*Number of First Jobs*	6,643,516	9,142,393	12,630,946	13,489,724	14,830,056
		*Number of Jobs*	6,987,301	9,920,339	15,099,242	16,810,611	19,378,263
Occupation	Trainee	*Average Real Wage*	7381	7449	7809	8540	8737
		*Number of First Jobs*	359,412	459,197	775,217	650,119	705,056
		*Number of Jobs*	394,058	558,196	1,005,564	997,419	1,092,714
	Blue-collar	*Average Real Wage*	15,565	15,994	13,927	12,139	11,892
		*Number of First Jobs*	4,139,724	5,448,182	7,290,505	7,507,121	8,336,025
		*Number of Jobs*	4,304,989	5,915,699	8,819,540	9,590,153	11,353,633
	White-collar	*Average Real Wage*	21,609	26,601	22,565	21,400	21,998
		*Number of First Jobs*	2,067,665	3,114,180	4,126,493	4,781,543	5,213,611
		*Number of Jobs*	2,208,584	3,319,380	4,785,366	5,626,490	6,298,740
	Manager	*Average Real Wage*	–	–	54,158	55,271	58,755
		*Number of First Jobs*	–	–	310,204	432,165	458,048
		*Number of Jobs*	–	–	347,185	467,760	505,339
	Executive	*Average Real Wage*	69,229	99,264	112,994	118,452	125,573
		*Number of First Jobs*	76,715	120,834	128,527	118,776	117,316
		*Number of Jobs*	79,670	127,064	141,587	128,789	127,837
Gender	Male	*Average Real Wage*	19,575	22,536	20,678	19,699	19,487
		*Number of First Jobs*	4,521,452	5,978,812	7,817,494	7,860,608	8,568,385
		*Number of Jobs*	4,725,507	6,474,057	9,309,306	9,690,644	11,156,455
	Female	*Average Real Wage*	13,554	15,607	13,983	13,421	13,557
		*Number of First Jobs*	2,122,064	3,163,581	4,813,452	5,629,116	6,261,671
		*Number of Jobs*	2,261,794	3,446,282	5,789,936	7,119,967	8,221,808
Geographical Area	South	*Average Real Wage*	14,626	16,885	15,385	12,545	12,493
		*Number of First Jobs*	998,473	1,320,880	2,012,641	2,209,947	2,445,527
		*Number of Jobs*	1,054,164	1,411,646	2,311,533	2,760,613	3,149,497
		*Number of Jobs*	448,716	672,132	1,015,887	1,190,746	1,278,266
	Center	*Average Real Wage*	18,065	20,919	18,342	16,770	16,666
		*Number of First Jobs*	1,291,519	1,781,923	2,465,992	2,772,321	3,089,940
		*Number of Jobs*	1,364,723	1,916,578	2,929,199	3,474,541	4,029,264
	North-West	*Average Real Wage*	19,597	22,684	20,330	19,985	19,946
		*Number of First Jobs*	2,568,139	3,241,840	4,209,843	4,353,810	4,739,277
		*Number of Jobs*	2,681,517	3,503,732	5,077,166	5,364,486	6,170,386
	North-East	*Average Gross Wage*	16,967	18,747	17,459	17,746	17,561
		*Number of First Jobs*	1,366,117	2,182,235	3,057,714	3,206,845	3,565,032
		*Number of Jobs*	1,438,181	2,416,251	3,765,457	4,020,225	4,750,850
Age Cohort	Under 30	*Average Real Wage*	12,713	13,980	11,359	9067	8494
		*Number of First Jobs*	2,673,776	3,786,099	4,194,559	3,020,003	3,378,076
		*Number of Jobs*	2,876,618	4,226,286	5,389,661	4,235,625	5,088,250
	Adults 31–50	*Average Real Wage*	21,011	23,942	20,795	18,496	18,246
		*Number of First Jobs*	3,055,080	4,258,624	6,885,981	7,827,848	7,737,063
		*Number of Jobs*	3,175,961	4,543,798	8,001,183	9,518,095	9,881,949
	Over 50	*Average Real Wage*	21,239	27,662	26,841	23,552	23,898
		*Number of First Jobs*	914,660	1,097,670	1,550,406	2,641,873	3,714,917
		*Number of Jobs*	934,722	1,150,255	1,708,398	3,056,891	4,408,064

Such phases are intimately linked to a series of legislative changes that have interested the Italian labour market. Historically, the process of gradual wage suppression started during the 1983–1984 period, with the government coalition led by the Socialist Party, reducing by three points the alignment between wage and inflation, with the aim of keeping the latter under control, the so-called *San Valentino agreement*, that was signed between the Craxi government and two of the three main Italian trade unions, with the exclusion of CGIL (*Confederazione Generale Italiana del Lavoro*). This adjustment was object of a referendum launched in 1985, which saw the prevalence of consensus for freezing wage growth and keeping inflation under control.^6^

The abolition of automatic wage indexation to inflation was accompanied by numerous debates and rifts between trade unions and eventually was fully enforced after one decade, in 1992, when Italy was preparing its entry into the currency union, with an agreement between the Amato government and all three main unions. That year marked the beginning of a reconfiguration of the wage bargaining process, the so-called cooperation period (*periodo della concertazione*), and of the wage policy (*politica dei redditi*) undertaken by the succeeding government led by Ciampi.

Wage compression was successfully pursued, as shown by the 5-year percentage change in real wages (Fig. 2b), which from the peak in 1991, recording a 12% growth, reached a negative value of −0*.*5% in 1996. Negative wage growth became a dominant trait of the labour market onwards, with other two minima, the first in 2002, just one year after the entry in the common currency union*,* and the second in 2007 at the beginning of the Great Recession. Wage growth was then positive only during the two modest recovery years, 2016 and 2017, and turned again negative in 2018.

The pillar of these reforms, and the first in response to the EU institutions’ indications, was the *Pacchetto Treu* in 1997 that multiplied the possible types of contractual regulations, introducing temporary contracts and strengthening part-time ones (existing since 1984). The *Legge Biagi* followed in 2003, further increasing the number of contractual regulations, allowing for short employment duration and introducing de facto forms of mini-jobs by means of outsourcing contracts (co.co.co), project-based contracts (co.co.pro), occasional or intermittent contracts (*lavoro occasionale*, *lavoro accessorio*).

The following liberalization reform was the *Legge Fornero* in 2012, which weakened the effectiveness of labour protection instruments, further encouraged open-ended contracts and reformed the pension system increasing retirement age. Two years later in 2014 the *Jobs Act* was implemented, the last major labour market reform and final straw of the flexibilization process, meant at easing firing processes and abolishing restrictions for firms with more than 15 employees. The Jobs Act definitely suppressed the remaining protections from invalid dismissals and introduced a less rigid typology of contract with time varying protections for employees (*contratto a tutele crescenti*).

**Fig. 3:**
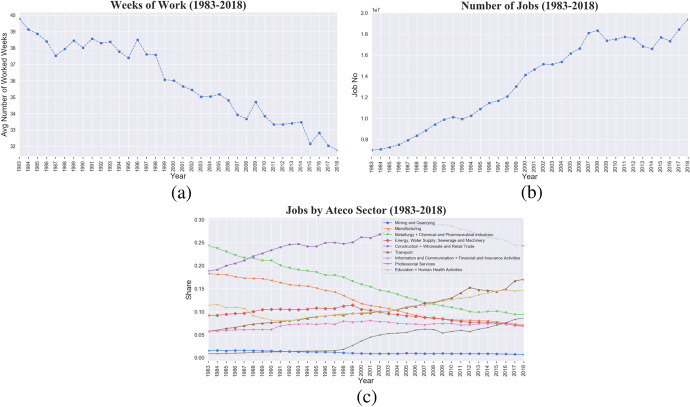
Italy, 1983–2018. Precarisation, fragmentation and deindustrialisation trends. **a** Average number of weeks of work in a year. **b** Number of jobs. **c** Share of jobs by different aggregations of 1-digit Ateco industrial sectors (Mining and Quarrying; Manufacturing; Metallurgy + Chemical and Pharmaceutical industries; Energy, Water Supply, Sewerage and Machinery; Construction + Wholesale and Retail Trade; Transport; Information and Communication + Financial and Insurance Activities; Professional Services; Education + Human Health Activities; Other Services)

This series of reforms, which were supposed to foster employment growth, did not produce the expected effect (Fana et al. 2016) but instead weakened the innovative capabilities of Italian firms (Cetrulo et al. 2019; Reljic et al. 2021). Moreover, they successfully resulted into an evident contraction in the number of worked weeks, contrasted however by an increasing number of jobs, as shown in Fig. 3a, b. Inclusion of atypical contractual forms, absent in our dataset, would have even further exacerbated the picture. Such diverging trends hint at a strong fragmentation of employment contracts – the temporal units of working activity – which exploded in number but dramatically shortened in time.

Nevertheless, such increasing number of jobs is distinctly concentrated in specific sectors. Panel (c) of Fig. 3 documents the well-known process of deindustrialization, with shares in manufacturing (orange line), chemical, metallurgy and pharma (green line) strongly declining from respectively 0*.*20% and 0*.*25% of total private employment in 1983 to a share of approximately 10% in 2018. In contrast, a trend of servitization is clearly evident, with increasing shares of construction, wholesale and retail trade (purple line), transport (brown line), education and human health activity (yellow line), professional services (grey line), in total accounting for more than 50% of employees in 2018.

**Fig. 4:**
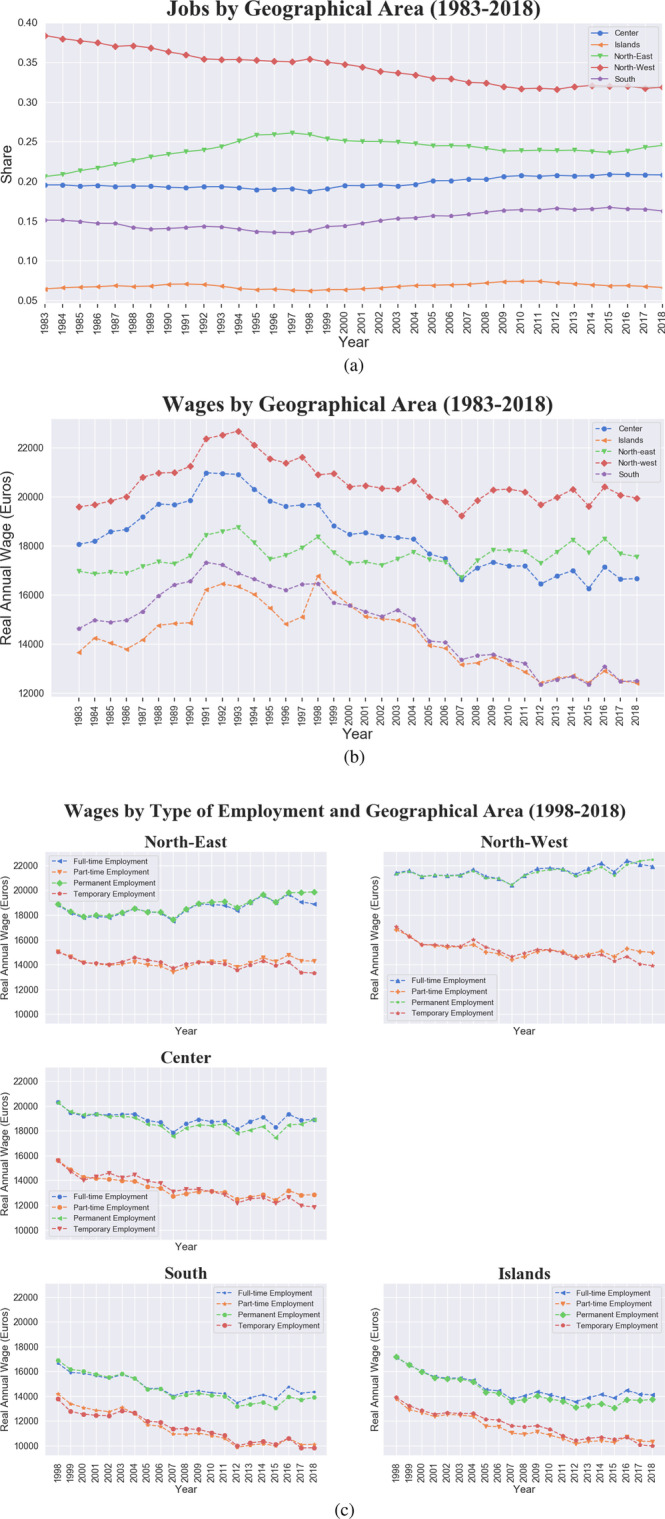
Italy, 1983–2018, geographical divergence trend. **a** Share of jobs by geographical area. **b** Real average wage by geographical macro-area (South, Islands, Center, North-East, North-West). **c** Real average wage by geographical macro-area and type of employment

Figure 4 presents the breakdown by geographical area, divided into North-East, North-West, South, Center and Islands. Job shares, shown in panel (a), remain roughly constant, with the exception of the North-West declining shares and the slightly increasing shares in the South. However, the increasing share of jobs in the South corresponds to the introduction of temporary jobs in 1998. Furthermore, in panel (b) we detect a marked pattern of wage divergence accelerating since 1998, a rebound of the North-East since 2007, and a huge decline in the South and Islands, with average wages ranging from 20 thousand euros in the North to 12 thousand in the South and Islands. The geographical wage gap however is also marked within the same geographical area between temporary/part-time and permanent/full-time jobs, as shown in panel (c). The lowest gap is found for the Islands, the highest is instead registered in the North-West, antipodal areas in terms of wage levels.

**Fig. 5:**
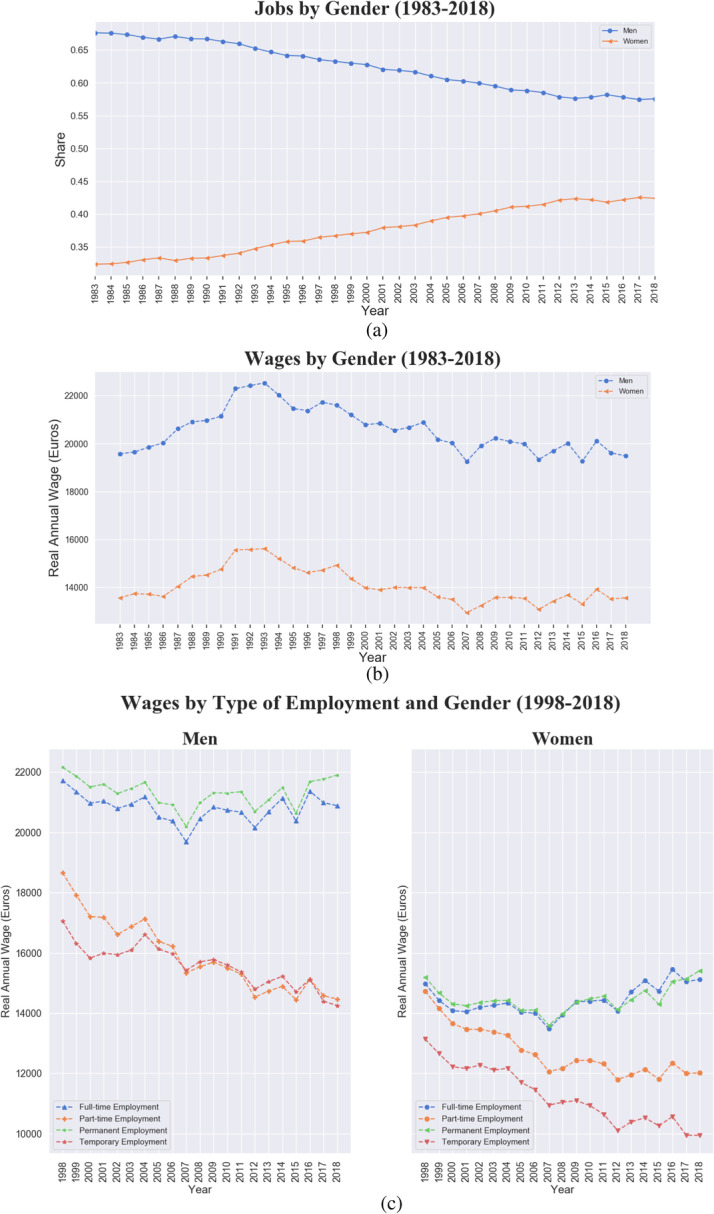
Italy, 1983–2018, feminization of the labour force and gender-pay gap. **a** Share of jobs by gender. **b** Real average wage by gender (Women in orange, Men in blue). **c** Real average wage by gender and type of employment

A growing participation in the labour market from the female component is visible in panel (a) of Fig. 5, with a share raising from 35% in 1983 up to 42% in 2018. However, the increasing demand of female jobs did not mapped into increasing real wages: the latter remain almost flat in real terms and the gender-pay gap (of approximately 6 thousand euros) does not show any contraction in the period of analysis. When looking at the wage dynamics by gender and employment type, a declining trend is recorded in temporary/part-time jobs for male and female jobs, albeit with temporary female jobs experiencing the lowest remuneration across all categories.

**Fig. 6:**
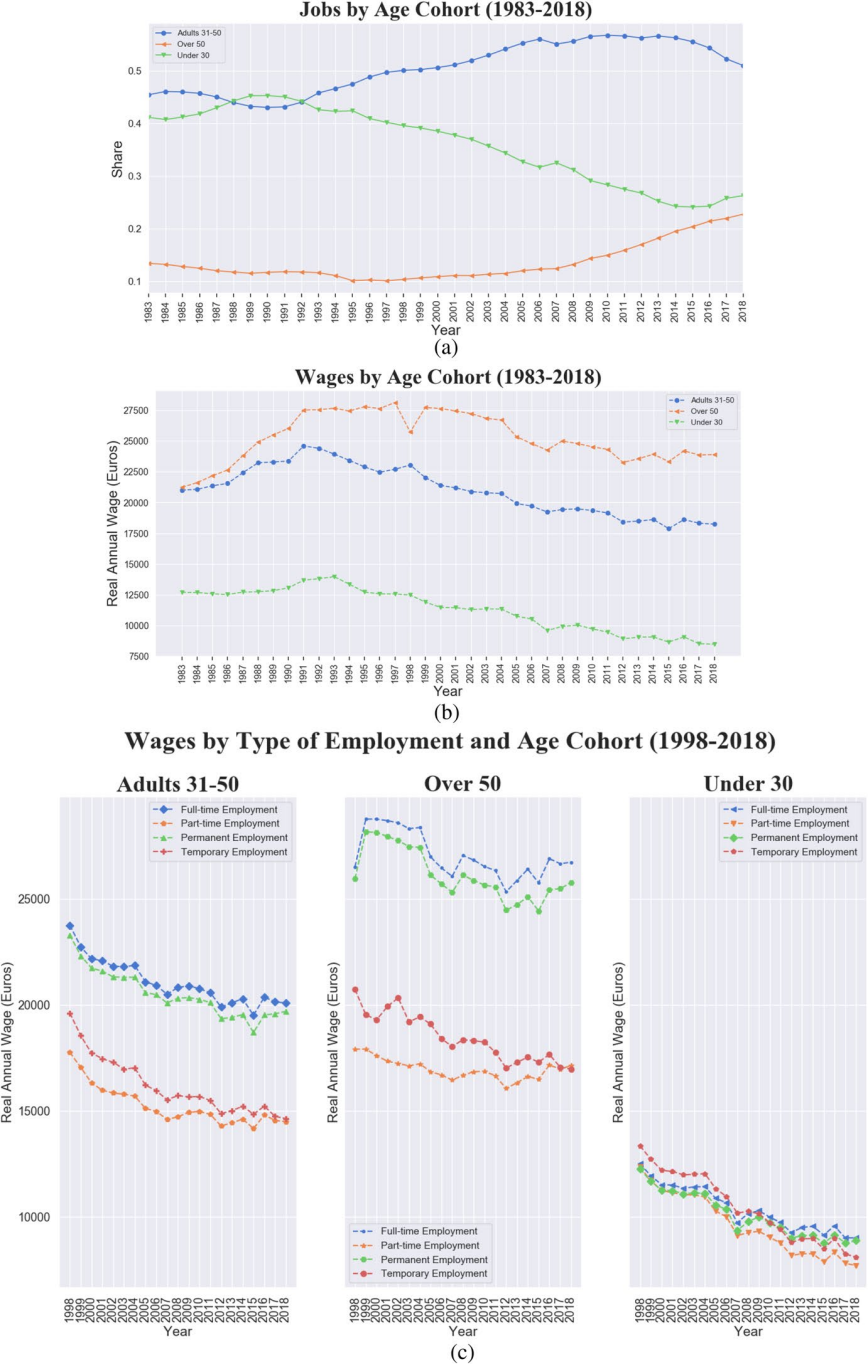
Italy, 1983–2018, ageing labour force trend. **a** Share of jobs by age cohort. **b** Real average wage by age cohort (Young under 30, Adults between 31 and 50, and Over 50). **c** Real average wage by age cohort and type of employment

Moving to the ageing labour market trend, Fig. 6a shows an increasing share of jobs for 31–50 years old workers since 1992, and a corresponding declining trend in the share of jobs performed by workers under 30. Although such a trend might also be due to a higher education rate raising the entry age in the labour market, since 1998 the growing fraction of jobs performed by workers over 50, that increasingly populated the labour market, appears quite alarming and also reflects an entry in the labour market with atypical contractual forms. The older segment also enjoys remarkable wage premium, as shown in panel (b). If the wage-age premium is not surprising, what is worrying is the declining remuneration of under 30s, which in 2018 earn on average less than 10 thousand euros per year. Panel (c) presents the breakdown of the three age cohorts by type of contract: while the wage gap between full-time/permanent contracts and part-time/temporary ones is visible for the two older cohorts, the remunerations of workers under 30, independently from their contract type, record a steep and monotonic convergence to the bottom.

**Fig. 7:**
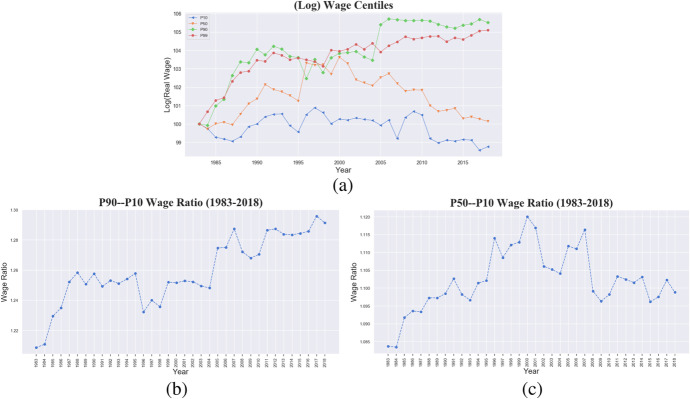
Italy, 1983–2018. **a** Real average wage centiles over time. **b** P90-P10 and **(c)** P50-P10 wage ratios

The observed patterns of wage divergence are mirrored by the movement of the synthetic wage inequality indicators presented in Fig. 7a which displays the time evolution of real wages in the 10th, 50th, 90th and 99th percentiles, with 1983 as the base year. While both bottom and median percentiles decline, the two top percentiles show clear increasing trends with a decreasing distance between P99 and P90 after 2005 and a growing distance from P50 and P10. Panel (b) presents the 90–10 wage percentile ratio: a visible and steep increasing trend is reported documenting divergence toward the top. Convergence towards the bottom between the 50th and 10th percentile is shown in panel (c), providing evidence in favour of a ‘proletarization’ hypothesis since 2000.

**Fig. 8:**
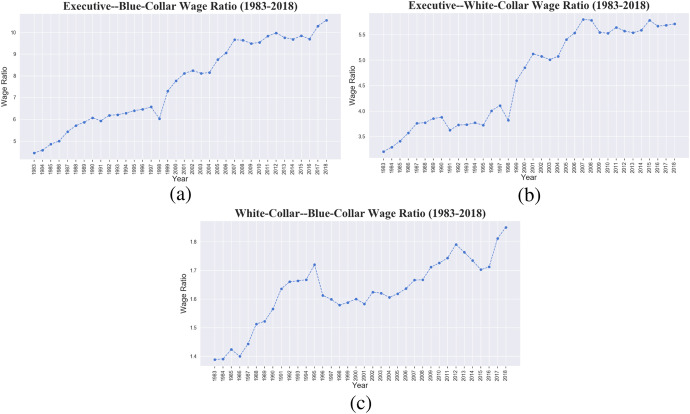
Italy, 1983–2018. **a** Executive-Blue Collar wage ratio. **b** Executive-White Collar wage ratio. **c** White Collar-Blue Collar ratio

Is the divergence at the top and convergence towards the bottom linked to the wages of different occupational categories? Figure 8 presents the dynamics of the wage gap between executives and blue-collars (panel a), executives and white-collars (panel b), white- and blue-collars (panel c). The latter statistics, which are generically not used as proxy of the inequality dynamics, give us a glimpse of the role of occupational categories in affecting wage inequality and indirectly on the relative bargaining power of the bottom occupational classes vis-à-vis the top ones. A clear pattern of lost bargaining power of blue- and white-collars over executives’ earnings appears, with the executive average pay increasing over time from less than 5 to more than 10 times that of blue-collars, and from 3 to 5.5 times that of white-collars. The white- vs blue-collar ratio presents by far a more compressed increasing trend, ranging from 1.4 to 1.8 times. However, combining this trend in inequality with information from Table 3 it emerges that the gap does not increase because of growing white-collar wages, which instead are more or less stable, but because of declining blue- versus white-collar wages.

Overall, together with a wage compression and job fragmentation history, we have documented patterns of divergence deriving from many alternative sources, namely geographical origin, age, gender and occupational divides. Our results confirm both the analysis provided by Rosolia (2010) on the longitudinal INPS database (WHIP) over the period 1985–2004, where the strong relation between socio-demographic characteristics and income gaps emerges; and the study by Bloise et al. (2018) that, using individual INPS LOSAI data over the period 1985–2014, observe not only a general increase in inequality but also a rising polarization across groups, in particular for what concerns the distance between top and bottom income earners.

Given the evidence presented so far, we have detected a strong influence exerted by socio-demographic characteristics on patterns of wage divergence, however without a conclusive understanding of the role played by each one of them. In the following we attempt to address such a task.

## Sources of wage inequality

While in the above we have observed a clear trend of increasing wage inequality, especially the two processes of divergence at the top and convergence at the bottom, in this section we explore the determinants of wage inequality by distinguishing the role played by the gender, age, geographical area and occupational category of the workforce. In order to do so, firstly we present an a priori decomposition exercise in Subsection 5.1, secondly, we propose a regression-based analysis in Subsection 5.2, and thirdly in Subsection 5.3 we study the association between negative wage episodes and our inequality determinants. Finally, Subsection 5.4 discusses our findings vis-à-vis the routinization hypothesis.

### Inequality decomposition: within and between components

With the aim of appreciating different characteristics of the overall, within- and between-group inequality distribution, we compare the trends of a number of inequality indicators, each of which is particularly sensible to specific and distinct features of the earnings distribution. In particular we focus on the Gini coefficient and different Generalised Entropy indices (*GE*(*α*)). For a population of *n* individuals and a discrete wage distribution **y** $\boldsymbol{\in}{\mathbb{R}}_{+}^{\boldsymbol{n}}$, where each worker has wage *y*_*i*_*,* (*i* = 1*,*...*,n*) and wages are indexed in non-decreasing order (*y*_*i*_ ≤ *y*_*i* + 1_), the Gini coefficient formulation we employ is defined as follows:

1 $$G=\frac{n+1}{n}-\frac{2}{n\sum\limits_{i=1}^n{y}_i}\left(\sum\limits_{i=1}^n\left(n+1-i\right){y}_i\right)$$

*G* = 0 in the case of perfect equality, i.e. when all individuals have the same wage, and *G* = 1 in a situation of maximum inequality, i.e. when a single individual earns the totality of wages. The Gini coefficient tends to be more sensitive to wage differences around the mode than in the lower or higher tails of the distribution (Green et al. 1994).

Concerning the Generalised Entropy indices we focus on *GE*(*α*) with *α* ∈ [0*,*1*,*2], where *GE*(0), *GE*(1), *GE*(2) correspond respectively to the Mean Logarithmic Deviation (MLD), the Theil Index and the Half Square of the Coefficient of Variaton (1/2 SCV).

Mathematically, considering a population of *n* individuals, with wage *y*_*i*_ (*i* = 1*,*...*,n*), arithmetic mean wage *m*, sample weight, if present, equal to *w*_*i*_, with *f*_*i*_ = *w*_*i*_*/N* and *N* =$\sum{w_i}$ (*N* = *n* when *w*_*i*_ = 1), these widely used inequality indicators are defined as follows:

2 $$GE\left(\alpha \right)=\left\{\begin{array}{cc}\frac{1}{\alpha \left(\alpha -1\right)}{\sum}_{i=1}^n{f}_i\left[{\left(\frac{y_i}{\overline{m}}\right)}^{\alpha }-1\right],& \alpha \ne 0,1\\ {}\frac{1}{n}{\sum}_{i=1}^n{f}_i\frac{y_i}{\overline{m}}\ln \frac{y_i}{\overline{m}},& \alpha =1,\\ {}\frac{1}{n}{\sum}_{i=1}^n{f}_i\ln \frac{m}{\overline{y_i}},& \alpha =0\end{array}\right.$$

where *α* is a real parameter that regulates the weight given to the distance between each individual’s wage and the average. For large values of *α*, *GE*(*α*) is particularly sensitive to wage differences at the top of the distribution, by contrast for small *α* it responds more to inequality at the bottom of the distribution (Jenkins 1995, 2009). *GE*(*α*) = 0 in the case of complete equality, while larger real values of the index indicate higher inequality in the distribution.

**Fig. 9:**
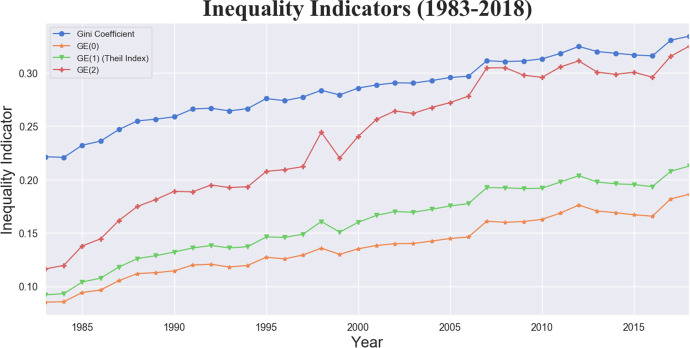
The Gini Coefficient and the selected general entropy indicators *GE*(*α*)*, α* ∈ [0*,*1*,*2], trends over the period 1983–2018

A distinct positive growth of inequality is shown by all indicators taken into consideration, albeit at different paces, as implied by their underlying mathematical and parametric constructions (Fig. 9). Table 3 presents their point values in 1983, 1993, 2003, 2013 and 2018, the starting years of each decade plus the last observation in our data-set. Table 4 instead displays their percentage variations in the time intervals 1983–1993, 1993–2003, 2003–2013, 2013–2018 and over the entire observation window. The change is much higher for all GEs (> +110%) rather than for Gini, that, being more sensitive to changes in the middle of the distribution, increases “only” by 50%. The highest percent changes are recorded for GE(1) and GE(2), thus confirming that inequality at the top of the distribution has increased the most over our time window. The greatest change is however recorded in the first decade (1983–1993) for all indicators.

**Table 3:** Values multiplied by 1000 of the Gini Coefficient and the selected general entropy indicators *GE*(*α*)*, α* ∈ [0*,*1*,*2], in 1983, 1993, 2003, 2013 and 2018

Year	Gini	MLD (GE(0))	Theil (GE(1))	1/2 SCV (GE(2))
1983	221	85	92	116
1993	264	118	135	192
2003	290	140	169	261
2013	319	170	197	300
2018	334	186	212	324

**Table 4:** Percentage variation of the Gini Coefficient and the selected general entropy indicators *GE*(*α*)*, α* ∈ [0*,*1*,*2], in the time intervals 1983–1993, 1993–2003, 2003–2013, 2013–2018 and over the entire time window 1983–2018

Time interval	Gini	MLD (GE(0))	Theil (GE(1))	1/2 SCV (GE(2))
1983–1993	19	39	46	65
1993–2003	9	19	25	36
2003–2013	1	21	16	15
2013–2018	5	9	8	8
1983–2018	52	119	130	179

Next, we develop a decomposition analysis of inequality by four workforce sub-groups defined by the occupational category, gender, age and geographic area of origin of each considered segment. We refrain from using the contractual regulation types, whether part-time or temporary, since for each category we do not have direct attribution but percentage of workers with a specific type of job contract. Alternatively, we should have ex-ante imputed to each category a type of job contract, according to a principle of prevalence (e.g., average blue-collar women in Piedmont beyond fifty, the totality of whom considered as full-time workers, while average white-collar women in Lombardy in the service sector beyond thirty all considered temporary workers). Given the within-category variability, although investigated, prevented us from operationalising this sort of attribution by prevalence.

In order to detect whether the overall change in inequality derives from changes within or between each sub-group, we rely on the decomposition method developed by Jenkins (1995), who in turn built on the seminal work of Shorrocks (1982, 1984). Similar analyses have been conducted for Italy by Franzini and Raitano (2019) who looked at income inequality and by Raitano (2021) who instead focused on wage inequality only.

The four attributes on which we base our workforce partitions are deeply important for understanding the overall dynamics of wage inequality. According to Goldthorpe (2002)’s class schema, occupational categories open the possibility to grasp the role played by class position in wage differentials. According to Wright (1979), to study income distribution we must look at the social structure of an economic system, and therefore focus on social classes which are shaped by social relations of production. In this sense, occupations and social classes are closely linked since the former are essentially defined by technical relations of production, while the latter embody the totality of social relations of production (accounting for the technical, authority and exploitation dimension). The gender factor takes into account the increasing relevance of female employment, occupational segregation and gender-wage gap (Bettio et al. 2013). Age is associated with different career prospects and degrees of job stability with a distinct impact on the wage level (Rosolia and Torrini 2007). The geography of wage inequality in Italy is linked to a profound North-South divide, characterised by strong labour market segmentation and differences in industrial structure (Sbardella et al. 2021) and infrastructures (Viesti 2021).

To carry out the decomposition exercise, we focus exclusively on the generalised entropy indices as they are easily decomposable across population groups. For the sake of brevity but with the aim of grasping differences both at the top and the bottom of the wage distribution, we show our results only for the Mean Logarithmic Deviation GE(0) and the Half Square of the Coefficient of Variation GE(2).

If individuals (employment relationships in our case) are grouped in a mutually exclusive and exhaustive way, inequality can be separated into a *within-group* component – the weighted sum of the inequalities in each group – and a *between-group* component – computed assuming that each employment relation’s wage corresponds to its group’s average income. Therefore, following Jenkins (1995), if we consider that the population is divided into *m* groups, *g*_1_*,g*_2_*,...,g*_*m*_, each with *n*_*k*_ individuals with *k* = 1*,...,m*, then *GE*(*α*) can be rewritten as:

3 $$GE\left(\alpha \right)={GE}^W\left(\alpha \right)+{GE}^B\left(\alpha \right)$$

where *GE*^*W*^ (*α*) is the within group inequality and *GE*^*B*^(*α*) is the between group inequality.

Looking in particular at the GE(0) and GE(2) Equations, we can write:

4 $${\displaystyle \begin{array}{cc} GE(0)= GE(0)^W+GE(0)^B=\sum_k^m v_k GE(0)^{(k)} + \sum_k^m v_k \log(1/s_k)\\ GE(2)= GE(2)^W+GE(2)^B=\sum_k^m v_k s_k^2 GE(2)^{(k)} + \sum_k^m v_k[s_k^2-1] \end{array}}$$

where *v*_*k*_ =$\frac{n_k}{n}$ is the population share of group $k,{s}_k=\frac{y_k}{\overline{y}}$ is the ratio of the average group wage to overall average wage, *GE*(*α*)^(*k*)^ (*α* = 0*,*2) is the inequality index for each group *k* and accounts for the inequality between the members of the group, that is assumed to be a separate population from the other groups.

Table 5 presents the decomposition analysis accounting for the *between* and the *within* components of overall wage inequality. While we report the results for some selected time windows, the decomposition exercise has been consistently replicated over the entire time period. Thanks to such decomposition, we are able to study the magnitude of the within and between group components.

**Table 5:** GE(0) and GE(2) within group and between group inequality in 1983, 1993, 2003, 2013 and 2018 (values multiplied by 1000)

Group (k)	GE(α)	1983	1993	2003	2013	2018
Gender	*GE(0) Within*	71	103	123	153	170
	*GE(0) Between*	14	15	17	17	16
	*GE(2) Within*	103	179	245	284	310
	*GE(2) Between*	13	13	16	16	14
Age cohort	*GE(0) Within*	55	79	91	116	119
	*GE(0) Between*	30	39	49	54	67
	*GE(2) Within*	89	156	218	257	271
	*GE(2) Between*	27	36	43	43	53
Geographical area	*GE(0) Within*	78	111	134	156	172
	*GE(0) Between*	7	7	6	14	14
	*GE(2) Within*	110	186	256	287	311
	*GE(2) Between*	6	6	5	13	13
Occupational category	*GE(0) Within*	39	39	43	60	70
	*GE(0) Between*	46	79	97	110	116
	*GE(2) Within*	45	52	51	53	63
	*GE(2) Between*	71	140	210	247	261

Among our partitions, occupational categories constitute the group characterised by the highest between component, which explains most of the overall inequality both using the Mean Logarithmic Deviation and the Half Square Coefficient of Variation, consistently with Raitano (2021). The trend is increasing over time, meaning that the degree of wage inequality has been rising across occupations; in addition, our proxy of social class constitutes the only partition where the between component overcomes the within component over the entire time period. Indeed, our class schema results to be very effective in explaining wage inequality and is more relevant than the other workforce characteristics.

This finding confirms that considering class position in terms of occupational hierarchies allows to account for a not negligible degree of wage inequality, as underlined for instance by Quintano et al. (2009) and Albertini (2013) for Italy and by Penissat et al. (2020) for European countries. This is true despite the fact that our occupational category group is based on a very broad classification, able to distinguish among only five types of occupations. Admittedly, it is not directly comparable with more refined and accurate socio-economic models that exploit higher degrees of disaggregation (Goldthorpe 2002; Weeden and Grusky 2005), that often include additional factors linked to the work process, such as the degree of autonomy and power exercised along segments of organizations (Wright 1998, 2000), or account for the role of social capital (Savage 2015). The importance of socio-economic groups and the occurrence of a distributional shift in favour of specific groups such as middle managers and executives have been indeed assessed in the literature (Brandolini et al. 2018), however without placing the issue of class inequality at the center of the analysis.

Differently from what happens for occupational categories, within group inequality prevails in the other partitions. In the case of gender this suggests that, despite the presence of a distinct gender-wage gap, stronger patterns of wage inequality can be found within the female and male groups rather than among women vs men. Geographical areas and age cohorts exhibit increasing degrees of within inequality that are systematically higher than the between component.

### Regression-based inequality decomposition

Two methods to decompose inequality metrics have been proposed in the literature, the a priori decomposition presented above and the *regression* approach. As discussed by Cowell and Fiorio (2011) these, rather than being alternative approaches, may be regarded as complementary. Both methods do not provide causal interpretations, but are however useful to get a clear picture of the degree of inequality within and between groups, and the role played by each factor characterising the groups in explaining the level of inequality. To precisely estimate the relative contribution played by each considered job characteristic in explaining the level of wage inequality, here we carry out a regression based inequality decomposition.

We rely on Fields’ regression decomposition method (Fields and Yoo 2000; Fields 2003) that, also thanks to its flexibility, has been widely adopted in the inequality literature. For instance, O’Donoghue et al. (2018) use a Fields’ method to study the impact of several variables on wage inequality in Ireland during the Great Recession; Manna and Regoli (2012) focus on Italian households income and wealth over the period 1998–2008; and Wan and Zhou (2005) study the determinants of income inequality in rural China showing that geography and capital inputs have the highest explanatory power.

By following Fields (2003), as a first step, to carry out the regression-based inequality decomposition let us consider a wage generating function for a population of *n* wage recipients and *k* determinants of inequality:

5 $${\displaystyle \begin{array}{c}\mathit{\log}\left({y}_i\right)=a{Z}_i\\ {}a=\left[\alpha \kern0.5em {\beta}_1\kern0.5em \begin{array}{ccc}{\beta}_2& \dots & \begin{array}{cc}{\beta}_k& 1\end{array}\end{array}\right]\\ {}{Z}_i=\left[\begin{array}{ccc}1& {x}_{i1}& \begin{array}{ccc}{x}_{i2}& \dots & \begin{array}{cc}{x}_{ik}& {\epsilon}_{\textrm{i}}\end{array}\end{array}\end{array}\right]\end{array}}$$

where *y*_*i*_ (*i* = 1*,...,n*) denotes the wage of employee *i*, *x*_*ij*_ (*j* = 1*,...,k*) the j-th explanatory variable, *β*_*j*_ its coefficient and ε_*i*_ the error term. After some transformations, it is possible to define the share of the wage log-variance (the relative factor inequality weight) that can be attributed to the *j*-th explanatory factor *s*_*j*_ as follows:

6 $${s}_j\left(\mathit{\log}(y)\right)=\frac{\mathit{\operatorname{cov}}\left[{a}_j{Z}_j,\mathit{\log}(y)\right]}{\sigma^2\left(\mathit{\log}(y)\right)}$$

where *σ*^2^(*log*(*y*)) is the variance of the dependent variable and *cov*[*a*_*j*_*Z*_*j*_*,log*(*y*)] is the covariance between the *j*-th explanatory factor and the dependent variable. If *s*_*j*_ *>* 0 the contribution of factor *x*_*j*_ increases inequality, while it decreases inequality for *s*_*j*_ *<* 0. Moreover, when the residual ε_*i*_ is excluded from *Z*, the sum of all relative factor inequality weights is equal exactly to *R*^2^(*log*(*y*)).

Therefore, for each year, we run a regression of the log yearly wages as a linear function of the variables that characterise our sub-groups of employment relations: gender (the base group being male employees), age cohort (the base group being employees under 30), geographical area (the base group being employees situated in Southern Italy), occupation (the base group being blue-collars). Such formulation of the wage generating function assumes that covariates are uncorrelated among them. For ease of readability, we present the results of the regression decomposition only for the five years considered in the previous exercise (1983, 1993, 2003, 2013 and 2018), but they are consistent across the whole 1983–2018 time window. The estimation of the coefficients is performed via OLS.

To distinguish covariates on the basis of their importance in explaining inequality, we focus on the factor inequality weights reported in Table 6. According to estimation results presented in the Appendix (Tables 8, 9, 10, 11, 12, 13, 14, 15, 16, 17 and Fig. 13), all variables have statistically significant coefficients, confirming their importance as wage distribution determinants. This is also confirmed by the fact that in our observation period not only the residual shows low values, always below 9%, but its magnitude is also decreasing over time, going as low as 2.4% in 2013 and 2.5% in 2018, indicating the growing relevance of our determinants.

**Table 6:** Factor inequality weights (%) for each considered variable across the entire workforce from Fields’ wage inequality regression decomposition

Factor	1983	1993	2003	2013	2018
Residual	8.60	5	4.3	2.4	2.5
Female	14.70	11.80	14	14	12.1
White collars	14.90	28.50	22.6	24.7	25.30
Executive	12.80	16.40	14.1	10.9	8.9
Trainees	13.90	12.10	4.4	−1.4	−1.9
Middle managers	–	–	14.40	17.10	15,90
30–50 years old	20.50	15.50	15	13	12.8
Over 50 years old	4.80	5.70	8.7	12.3	18.1
Central Italy	0.60	0.5	0	−0.3	−0.3
Islands	0.90	0.5	0.2	0.1	0.2
North Eastern Italy	−0.60	−1.1	−0.3	1.8	1.5
North Western Italy	8.80	5.10	2.6	5.4	4.8
Total	100	100	100	100	100

**Fig. 10:**
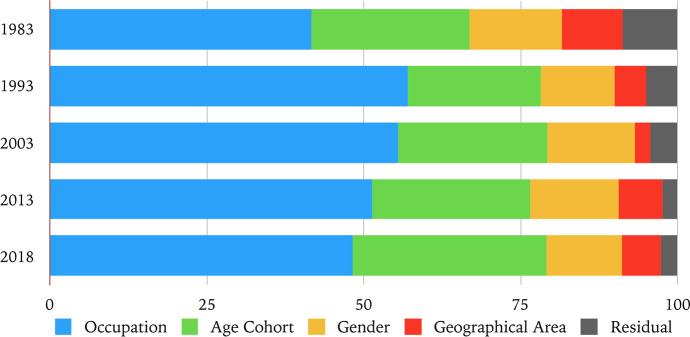
Total factor inequality weights (%) for each variable

The sum of occupational category factors displays the highest factor weights as inequality–enhancing (with respect to the base group of blue-collars) in each year. Occupational categories are then followed by the age and gender attributes, which seem to play a significant role in explaining inequality, whereas lower weights are associated to geographical areas. Over time we register the reducing weight of the middle-age fraction of employees and the increasing one of the older segment, the constant weight of female jobs and the increasing role played by white-collars and middle managers. Along the line of Fields (2003), to facilitate the interpretation of the factors and ease of visualisation, in Fig. 10 we sum up the relative contributions of each group components to verify which workforce partition contributes the most to inequality during the years under analysis. Consistently with the disaggregated weights, the occupational category group (in blue) plays the leading role in explaining inequality, followed by age cohort group (green) and gender (yellow).

### Wage losses by population sub-groups

Beyond the exploding inequality trend, the period under analysis records enormous wage losses in real terms (cf. Fig. 2). Here we investigate how these have manifested among the groups detailed in the previous analysis and which workforce partition has suffered the most.

In order to do so, firstly we attribute a panel structure to our dataset, whereby the repeated observation corresponds to a category given by the combination of gender (2 types), age class (3 types), region (20 types) and occupational category (5 types). Then, for each combined category we compute the annual negative wage growth episodes. The objective of this analysis is to detect the extent to which such categorical attributes are correlated with the event of a wage loss and if the observed differences across groups are statistically significant, signalling a stratification of the event.

Table 7 presents for five periods (1983–1984, 1993–1994, 2003–2004, 2013–2014, 2017–2018) the occurrence of a yearly wage loss event according to each different group, both showing the total number and the percentage of jobs affected by a wage loss. To check for the presence of a possible conditional dependence, we compute the Pearson’s *χ*^2^ statistic and the *χ*^2^ likelihood ratio, and test whether the distribution of the events across population groups is independent from the partitions.^7^ Both tests look at the differences between observed and theoretical frequencies under the null hypothesis that variables manifest independently, and therefore differences between theoretical and empirical frequencies are not statistically significant. While the Pearson *χ*^2^ test computes the squared difference between them, the likelihood ratio *χ*^2^ computes the ratio of the two.

**Table 7:** Wage loss distribution by population sub-groups

			1983–1984		1993–1994		2003–2004		2013–2014		2017–2018	
			*Wage Loss*	*No Wage loss*	*Wage Loss*	*No Wage loss*	*Wage Loss*	*No Wage loss*	*Wage Loss*	*No Wage loss*	*Wage Loss*	*No Wage loss*
Gender	*Female*	Total	817,103	1,471,117	3,496,842	118,505	4,506,501	1,469,782	2,183,352	4,816,306	3,696,829	4,524,884
		%	36	64	97	3	75	25	31	69	45	55
	*Male*	Total	2,376,143	2,391,379	5,902,912	723,078	2,934,370	6,427,113	3,174,910	6,411,931	7,054,192	4,102,214
		%	50	50	89	11	31	69	33	67	63	37
Age class	*Under 30 years old*	Total	1,640,055	1,234,317	4,273,143	57,99	2,927,300	2,342,289	1,867,241	2,148,484	3,523,683	1,564,492
		%	57	43	99	1	56	44	46	54	69	31
	*31–50 years old*	Total	1,421,036	1,829,821	4,224,020	552,695	3,712,132	4,596,363	2,686,362	6,652,674	5,364,089	4,517,860
		%	44	56	88	12	45	55	29	71	54	46
	*Over 50 years old*	Total	132,155	798,358	902,591	230,898	801,439	958,263	804.659	2,427,079	1,863,249	2,544,746
		%	14	86	80	20	46	54	25	75	42	58
Geographical area	*Central Italy*	Total	562,762	815,880	1,934,602	29,473	1,908,525	1,098,807	1,217,912	2,212,351	2,258,875	1,770,324
		%	41	59	98.5	1.5	63	37	36	64	56	44
	*Islands*	Total	140,347	323,134	565,226	96,683	783,073	269,602	571,891	581,682	886,419	391,847
		%	30	70	85	15	74	26	49	51	69	31
	*North-Eastern Italy*	Total	757,976	713.083	2,498,417	68,334	58,679	3,206,401	453,869	3,488,475	2,579,679	2,171,146
		%	52	48	97	3	2	98	12	88	54	46
	*North-Western Italy*	Total	1,524,013	1,155,180	3,365,941	252,823	2,770,665	2,353,877	1,560,438	3,759,135	3,495,838	2,674,548
		%	57	43	93	7	54	46	29	71	57	43
	*Southern Italy*	Total	208,148	855,219	1,035,568	394,270	1,391,818	968.208	1,554,153	1,186,594	1,530,210	1,619,233
		%	20	80	72	28	59	41	57	43	49	51
Job class	*Blue collars*	Total	1,452,605	2,893,153	5,374,197	763,090	4,524,299	4,359,145	2,297,499	7,144,097	9,402,865	1,950,768
		%	33	67	88	12	51	49	24	76	83	17
	*White collars*	Total	1,386,681	853,159	3,389,947	28,508	2,565,243	2,319,362	2,852,288	2,731,902	1,219,095	5,079,645
		%	62	38	99	1	53	47	51	49	19	81
	*Executives*	Total	6,548	74,255	111,353	14,757	30,538	111,303	55,224	71,326	47,448	80,326
		%	8	92	88	12	22	78	44	56	37	63
	*Trainees*	Total	347,412	419,29	524,257	35,228	300,322	777.69	35,146	927,88	35,176	1,057,469
		%	89	11	94	6	28	72	4	96	3	97
	*Middle Managers*	Total					20,469	329,395	118,105	353,032	46,437	458,890
		%					6	94	25	75	10	90

Considering, e.g., the two-way frequency of female and male workers, a significant Pearson *χ*^2^ test implies that the difference between the distribution of a wage loss among female and male workers is significant. In all the years under analysis, we find strong evidence of dependence (we reject the null hypothesis of independence) thus implying that the probability of recording a wage loss is not independent from categorical attributes such as gender, age, geographical area and occupational category.

**Fig. 11:**
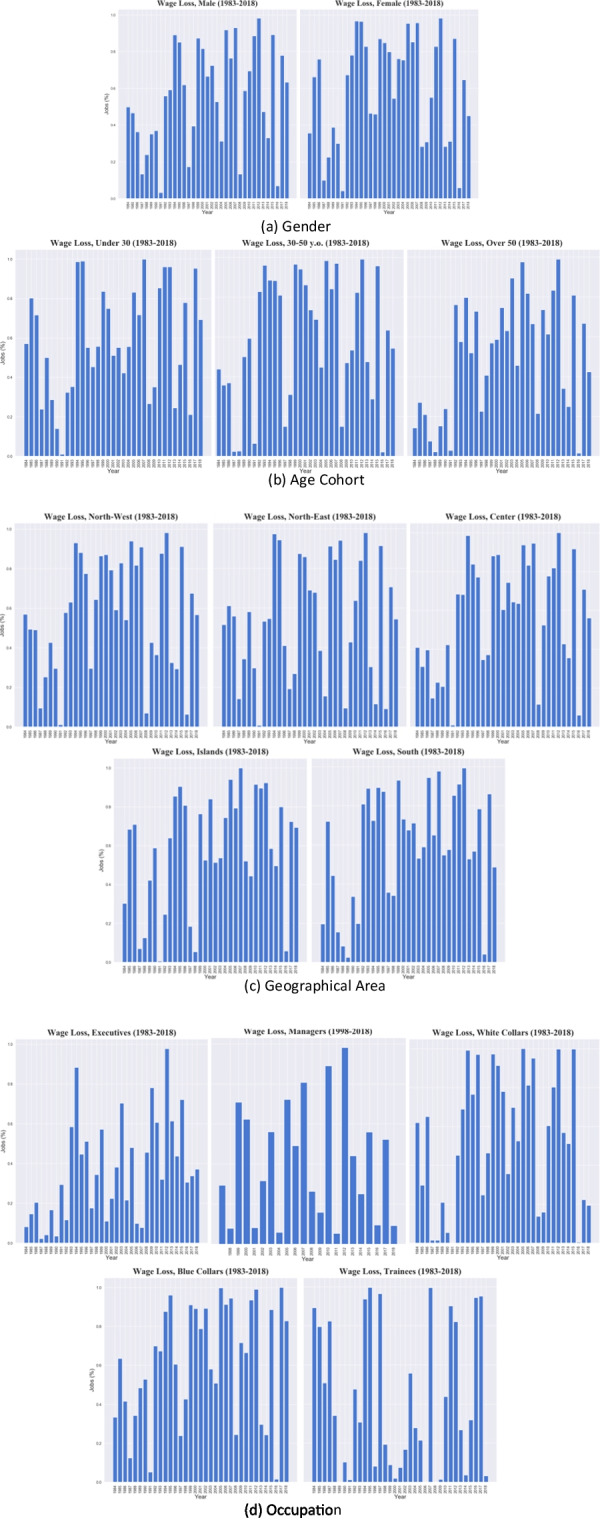
Wage loss events by population sub-groups

Figure 11 shows the distribution of wage loss events for each of the four determinants of inequality. Firstly, the event is not rare as in all years under analysis we find a marked presence of blue areas in the plots. Additionally, wage losses are more concentrated across female and youth jobs with respect to male and older jobs. Among the occupational category sub-groups, blue-collars and white-collars record the highest percentage of wage losses, whereas Northern regions are generically more resilient to loss events.

### Why wage inequality is not a matter of technology

So far we have focused on structural determinants but we have been silent about the role played by skills and technology, the two most prominent responsible of wage inequality according to mainstream economics: first the skill bias technical change (SBTC) (Acemoglu 2002; Katz and Murphy 1992; Autor et al. 1998) and afterwards the routine-biased technical change (RBTC) (Autor et al. 2003, 2006; Spitz-Oener 2006) approaches have become the dominant frameworks to explain inequality as a purely market problem. According to the SBTC approach, the rising wage inequality detected in the U.S. since the end of the 1970s is primarily due to an increasing demand for graduated vis-à-vis non-graduated workers. However, given the impossibility of explaining the growth in low skill jobs, a new variant of the “canonical model” was proposed, focusing the attention on the set of tasks embodied in each job activity rather than the skills and on the substitution effect of new technology on highly routinized activities (Acemoglu and Autor 2011; Autor and Dorn 2013). Therefore, the RBTC theory interpreted rising inequality as the result of the increasing adoption of computers, able to substitute human activities performing repetitive tasks – easily translatable into standards and codes – concentrated in the middle part of the occupational categories. Accordingly, the RBTC hypothesis predicts that middle jobs were at higher risk of being replaced by computers during the 2000s and by robots and artificial intelligence at present. As a consequence, middle-skilled workers would have shifted towards simpler manual or more complex abstract tasks, with an ensuing relative increase in the bottom and top tails of the distribution. The resulting occupational polarization due to human-replacing technologies should have therefore automatically turned into wage polarization, since wages are expected to closely follow job demand.

An enormous literature spurred in search of the evidence for such polarization, which empirically should have manifested into a U-shaped curve when looking both at employment and wage variations, as measured by wage percentiles (used as a proxy of skills) (Goos et al. 2009; Michaels et al. 2014). Thereafter, economic policies addressing inequality have generally been fully skill-oriented: taming inequality became a matter of education.

A strong critique to the latter theory has been put forward by an institutional approach to labour markets (Dosi et al. 2018; Mishel and Bivens 2021) and has been questioned even by more mainstream contributions (Stansbury and Summers 2020). A series of theoretical criticisms to this form of technological determinism includes: (i) a temporal mismatch between computer adoption and rising wage inequality (Card and DiNardo 2002); (ii) lack of evidence, even acknowledged by the proponents of the theory (Autor 2015a), of the purported U-shaped pattern in every decade since the end of 1970s in the U.S. (Mishel and Bivens 2017); (iii) contradictory results on the emergence of a job polarization pattern across European economies and presence of heterogeneity (Fernández-Macías and Hurley 2017); (iv) institutional features behind inequalities away from technology and instead related to macro-economic policies, structural change, international trade competition, labour market regulation (Mishel and Bivens 2021), work organisation (Holm et al. 2020), and firms’ strategy of outsourcing (Weil 2014); (v) a more comprehensive notion of wages and employment intended not simply as the result of demand and supply dynamics, but rather as the outcome of bargaining and conflicts; (vi) distinction between wages and skills level (usually proxied in terms of wage rank) empirically confirmed by declining college-wage premia, increasing number of underpaid over-skilled workers (Cappelli 2015) and growing inequality at the top of the income distribution (Atkinson et al. 2011; Mishel and Bivens 2021); (vii) lack of perfect substitutability between computers and routine tasks^8^; (viii) reductionist view of the labour activity in itself.^9^

Despite the criticisms that were raised, in this final part of our empirical analysis we perform a last exercise to detect the potential presence of polarization in the Italian economy and to check for the indirect role of technological change in the rising wage inequality we have detailed above. Therefore, by following Acemoglu and Autor (2011), we perform a locally weighted Gaussian-smoothing regression of changes in employment and wage shares by wage percentile rank (used in this context as a proxy for occupational skill). For each wage percentile *i* the employment share is defined as $\frac{E_{i,t}}{E_t}$, where *E*_*i,t*_ is total employment in percentile *i* and year *t*, and *E*_*t*_ is total employment in year *t*. For each starting year *t*_0_ we then plot the change $\left(\frac{E_{i,t}}{E_t}-\frac{E_{i,{t}_0}}{E_{t_0}}\right)$. The shares thus sum to one across percentiles $\left({\sum}_{i=0}^{100}\frac{E_{i,t}}{E_t}=\frac{E_t}{E_t}=1\right)$ and changes in shares sum to zero. A similar procedure is followed for wages.

**Fig. 12:**
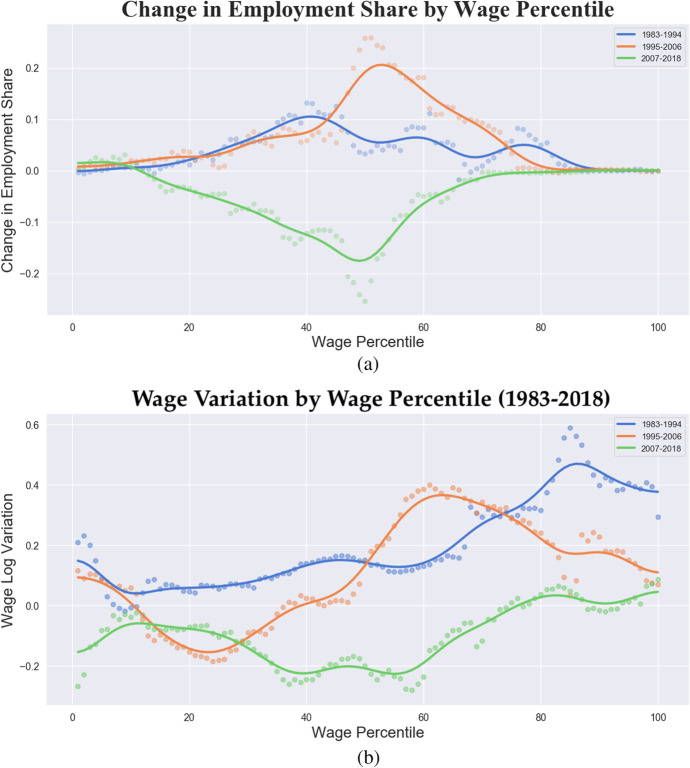
Italy, 1983–2018. **a** Employment polarization. The figure plots 10-year changes in employment shares by 1983 wage percentile rank (1983–1994 in blue, 1995–2006 in orange, 2007–2018 in green). **b** Wage polarization. Real average wage 10-year logarithmic change (1983–1994 in blue, 1995–2006 in orange, 2007–2018 in green)

**Fig. 13:**
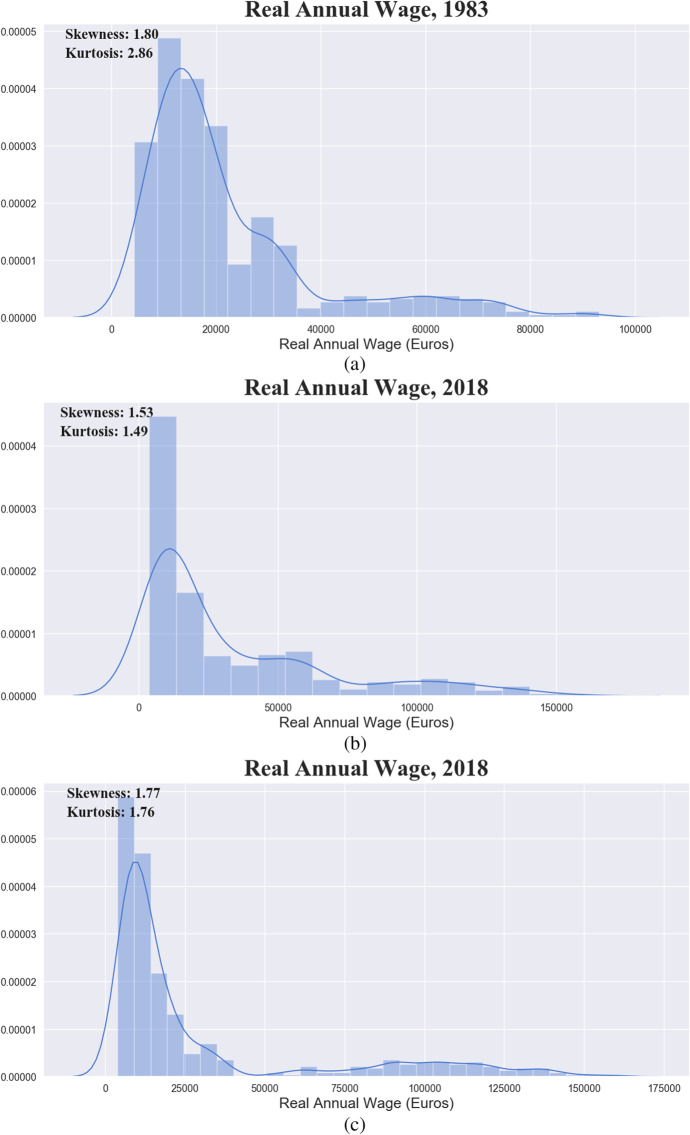
Italy, 1983–2018. **a** Real gross average wage distribution, Gaussian Kernel Density Estimation (KDE) in 1983; **b** Real gross average wage distribution, Gaussian KDE in 2018; **c** Real gross average wage distribution excluding executives, Gaussian KDE in 2018

Results are presented in Fig. 12. We replicate the analysis for three periods (1983–1994), (1995–2006), (2007–2018) and show changes in employment (panel (a)) and in wages (panel (b)) by 1983 (the starting year of our data-set) wage percentiles.

Firstly, no tout-court U-shaped pattern is present in employment changes: while in the previous two decades we do find evidence of a rather hump-shaped pattern (i.e., evidence of increases, and not decreases, in the middle part of the income distribution), in the last decade we record a decrease in the middle part, but with no increase neither in the lower nor in the upper part of the wage distribution, as predicted by the routine-bias technical change model. With reference to wage changes, we clearly see a gradual downward wage compression occurring over the almost four decades of analysis: while in the first period wages were rising relatively more for higher paid occupations, negative wage growth started to be recorded for low-paid occupations already in the period 1995–2006. A generalised negative wage growth is found along the entire wage distribution, except for wages at the very top in the last period.

Our results are indeed in line with Kristal and Cohen (2017) which find that much of rising U.S. wage inequality is due to labour dis-empowerment, represented by declining unionization and minimum wages, rather than technologies, and they echo the results in Dosi et al. (2021a) who show that increasing weights of deunionized firms have sparked both wage inequality and firm-level heterogeneity.

## Conclusions

By using administrative data on the Italian labour market, this paper has documented a series of medium-run trends within an overall picture of exploding inequality at the top and convergence toward the bottom, namely wage compression, servitization, job flexibilization and fragmentation, ageing labour force, feminization, geographical divergence. Among such potential determinants of inequality we have shown that the occupational category is the only factor presenting a higher between component vis-à-vis the within component, documenting that social classes, represented here by macro-occupational categories as intended by the early work of Sylos Labini (1974), are still the main determinant of divergence, notwithstanding the role played by age, gender and geographical divides.

The Italian PNRR (*Piano Nazionale per la Ripresa e la Resilienza*), aimed at promoting a period of deep transformation of the Italian economy by investing almost 200 billion euros in six years, has identified three main divergent patterns in the labour market which require urgent action, namely (i) generational asymmetries, (ii) gender asymmetries, (iii) geographical asymmetries. Although we acknowledge the need to tame the latter divergences, the elephant in the room is represented by occupational asymmetries that according to our findings are the root cause of the exploding 90–10 wage gap ratio, that is inequality at the top.

Wage inequality, we have shown, is an institutional result: it is largely due to wage compression strategies started with the period of cooperation (*periodo della concertazione*) in 1993 and perpetuated by a series of structural reforms aimed at making flexible the labour market in line with the neo-liberal orthodox consensus. Almost thirty years of such policies brought about a country marked by deep stratification processes, wherein social and economic risks, such as wage losses, are largely concentrated among young female blue-collars, in disadvantaged areas. Such stratification reverberates from the economic to the social dimension, with cumulation of income, occupational and safety risks that are fully on the shoulder of the very same most vulnerable categories (Cetrulo et al. 2020b, 2022).

Evidence of institutionally based roots of inequality, away from technological deterministic prediction of sheer polarization, has been recently put forward also by Mishel and Bivens (2021) who, by focusing on the U.S., strongly advocate for a rebalancing of labour power as the only effective redistributive policy measure to revert inequality trends:

Neither slow productivity growth nor inevitable economic forces can explain U.S. wage problems. Rather, wage suppression reflects the failure of economic growth to reach the vast majority. It was a “failure by design” (Bivens 2011), engineered by those with the most wealth and power. The dynamics are primarily located in the labor market and the strengthening of employers’ power relative to their rank-and-file workforce (which increasingly includes those workers with a four-year college degree). In other words, the dynamics that have challenged the growth of living standards for the vast majority are based on workers not sharing in economic gains [...]. (Mishel and Bivens, 2021, p. 2–3]

If from a policy perspective our paper stresses the role of redefining power relationship between top and middle-bottom occupational categories, from an analytical perspective, although suffering from the absence of individual longitudinal data, it puts under the spotlight the role played by social classes in understanding the ongoing labour market trends. This enlarges the scope of investigation of inequality and stratification and calls for a deep reconsideration of the role of occupations as main determinants of divergence, actually more relevant, and by far, than individual attributes as gender, age and geographical location.

Some limitations are however important to highlight. First, the very notion of social class is very complex to empirically operationalize. In general, whenever possible, the literature has used full-digit level disaggregation of occupational data, but it has also linked political and social attitudes (e.g., consumption habits) as determinants of social classes (Weeden and Grusky 2005). Our investigation lacks such detailed information, which however might also dilute in too many rivulets the identification of social classes. Without diminishing the fundamental role played by other sources of income in social classes analysis, such as rent from land and profit from capital, we do assume that for the majority of the working population the employment relation (and the corresponding wage) is the fundamental locus that actually determines the positioning of individuals over the social structure. Rather, we focus the attention on contradictory locations within the so-called “middle class” spectrum looking at the different positions covered by white-collars, middle managers and executives.

Second, the period under analysis is a hotbed of institutional changes with the national political agenda being shaped by the necessity of complying with the European so-called “external constraint” (Baccaro and D’Antoni 2020). Indeed, with the exit of Italy from EMS in 1992 and the urgency of entering back as soon as the fluctuations of lira were again under control, Italian governments put in place several policies in order to restore the competitiveness of the country. A strict control of the public debt was implemented, mainly through a massive reduction in public spending; the intervention of the State in the economy was downsized with the privatization of national companies owned by IRI such as Telecom and Alfa Romeo; the banking system was reformed (Graziani 1998). Third, such structural changes combined with the re-entering of the national currency into the EMS in 1996 clearly affected the overall productive structure of the country and influenced firms’ strategies (Landini et al. 2020). While big firms’ relevance was declining, industrial districts composed of small and interconnected firms were rising, also as the result of employers’ interest to restructure and delocalize “at local level” the production in order to weaken trade unions’ power and therefore relieve pressure on wage increases (Graziani 1998).

Considering that wage bargaining processes, or lack thereof, largely occur in workplaces, our results are silent about the workplace role in affecting inequality (Tomaskovic-Devey et al. 2020) and more broadly social classes, as they are about firm internal labour market policies and wage setting-schemes. Therefore, other confounding mechanisms, which we are not able to rule out from the analysis, might influence our results. These range from workplace-based management practices, job-offshoring of some productive functions adversely impacting on lower classes, deunionization and, more in general, transfer of firm-level productivity heterogeneity into wage-level heterogeneity by occupational categories. While the literature has recently advanced on the empirical identification of large productivity differentials across Italian firms (Calligaris et al. 2018; Dosi et al. 2021b) less has been done in terms of workplace wage differentials (intended as firm-level wages by occupational categories, see Avent-Holt et al. (2020)), beyond average ones (Cirillo and Ricci 2022) and only few attempts look at the role of deunionization in affecting firm-level wage inequality (Dosi et al. 2021a).

Future lines of research entail the understanding of the role played by sectoral and technological specialisation in affecting inequality trends at the regional level (Sbardella et al. 2017) in order to further explore the North-South divide, and more in general the role of geographic inequality deriving from different levels of innovation dynamism and agglomeration effects (Lee and Rodríguez-Pose 2013), but also analyses meant at tackling determinants of functional income inequality, studying, e.g., the dynamics of the labour share in the medium-run. In addition, our research remains silent about the actual motivation behind increasing managerial remuneration and power inside firms, and what are also the underlying benefits for capitalists in exacerbating hierarchical and positional asymmetries inside workplaces. The latter may represent a fruitful avenue for of future research delving into the unfolding of social classes.

## Data Availability

Data subject to third party restrictions.

## Appendix

### Regression-based inequality decomposition

**Total workforce**

**Table 8:** Econometric regression on log yearly wage in 1983

Log yearly wage 1983	Coef.	Std. Err.	t	P > |t|	[95% Conf. Interval]
Female	−.3360131	.0130721	−25.70	0.000	−.3617118 –.3103145
White collars	.3516309	.0132352	26.57	0.000	.3256115 .3776502
Executives	1.277227	.0560667	22.78	0.000	1.167004 1.387449
Trainees	−.4924298	.0271987	−18.10	0.000	−.5459001 –.4389595
30–50 years old	.354491	.0133622	26.53	0.000	.328222 .3807599
Over 50 years old	.3203695	.0192573	16.64	0.000	.2825114 .3582276
Central Italy	.2009248	.020298	9.90	0.000	.1610206 .2408289
Islands	−.0860071	.0278459	−3.09	0.002	−.1407498 –.0312644
North Eastern Italy	.2310217	.0200602	11.52	0.000	.1915851 .2704582
North Western Italy	.2999016	.0180021	16.66	0.000	.264511 .3352921
_cons	9.302424	.0181377	512.88	0.000	9.266767 9.338081
N	411				
Adjusted R-squared	0.9114				

**Table 9:** Regression-based decomposition of inequality on log yearly wage in 1983

Decomp.	100*s_f	S_f	100*m_f/m	CV_f	CV_f/CV(total)
Residual	8.6438	0.0036	−0.0000	−6.04e+14	−1.46e+16
Female	14.7076	0.0061	−1.1222	−1.4472	−34.8585
White collars	14.9002	0.0062	1.1468	1.4727	35.4740
Executives	12.8056	0.0053	0.1503	9.3228	224.5580
Trainees	13.9438	0.0058	−0.2865	−4.0954	−98.6463
30–50	20.5255	0.0085	1.6625	1.0968	26.4188
Over 50	4.8118	0.0020	0.4422	2.5478	61.3678
Centre	0.5774	0.0002	0.4049	2.0322	48.9505
Islands	0.9154	0.0004	−0.0570	−3.8220	−92.0593
North Eastern Italy	−0.5902	−0.0002	0.4906	1.9667	47.3715
North Western Italy	8.7591	0.0036	1.1875	1.2687	30.5596
Total	100.0000	0.0415	100.0000	0.0415	1.0000

**Table 10:** Econometric regression on log yearly wage in 1993

Log yearly wage 1993	Coef.	Std. Err.	t	P > |t|	[95% Conf. Interval]
Female	−.3608618	.0111256	−32.44	0.000	−.3827339 –.3389897
White collars	.5299623	.0113552	46.67	0.000	.5076387 .5522858
Executives	1.583107	.0459912	34.42	0.000	1.492692 1.673523
Trainees	−.5091363	.023455	−21.71	0.000	−.5552471 –.4630255
30–50 years old	.3401489	.011386	29.87	0.000	.3177649 .3625329
Over 50 years old	.3857652	.0174467	22.11	0.000	.3514662 .4200643
Central Italy	.1625902	.0179258	9.07	0.000	.1273494 .1978309
Islands	−.0705613	.0238629	−2.96	0.003	−.1174741 –.0236485
North Eastern Italy	.1654493	.0171409	9.65	0.000	.1317516 .199147
North Western Italy	.2467609	.0161534	15.28	0.000	.2150045 .2785173
_cons	9.393638	.0157269	597.30	0.000	9.36272 9.424555
N	410				
Adjusted R-squared	0.9488				

**Table 11:** Regression-based decomposition of inequality on log yearly wage in 1993

Decomp.	100*s_f	S_f	100*m_f/m	CV_f	CV_f/CV(total)
Residual	4.9922	0.0023	0.0000	9.60e+13	2.05e+15
Female	11.7914	0.0055	−1.2803	−1.3723	−29.3737
White collars	28.5416	0.0133	1.8110	1.4119	30.2218
Executives	16.3930	0.0077	0.2071	8.7899	188.1472
Trainees	12.0849	0.0056	−0.2926	−4.1004	−87.7686
30–50 years old	15.4713	0.0072	1.5911	1.0891	23.3124
Over 50 years old	5.7427	0.0027	0.4568	2.7646	59.1766
Central Italy	0.4820	0.0002	0.3208	2.0460	43.7954
Islands	0.4531	0.0002	−0.0488	−3.7139	−79.4962
North Eastern Italy	−1.0885	−0.0005	0.4115	1.7644	37.7679
North Western Italy	5.1363	0.0024	0.8901	1.3549	29.0023
Total	100.0000	0.0467	100.0000	0.0467	1.0000

**Table 12:** Econometric regression on log yearly wage in 2003

Log yearly wage 2003	Coef.	Std. Err.	t	P > |t|	[95% Conf. Interval]
Female	−.4090858	.0094812	−43.15	0.000	−.4277124 –.3904591
White collars	.5543072	.0101205	54.77	0.000	.5344245 .57419
Executives	1.8362	.0464718	39.51	0.000	1.744902 1.927499
Trainees	−.2180298	.0194036	−11.24	0.000	−.25615 –.1799096
Middle managers	1.191567	.0301086	39.58	0.000	1.132416 1.250718
30–50 years old	.3878121	.0103008	37.65	0.000	.3675753 .408049
Over 50 years old	.509484	.0157425	32.36	0.000	.4785563 .5404117
Central Italy	.0927542	.0152335	6.09	0.000	.0628265 .1226818
Islands	−.0436096	.0205359	−2.12	0.034	−.0839544 –.0032648
North Eastern Italy	.1372863	.0144755	9.48	0.000	.1088477 .165725
North Western Italy	.1754438	.0137834	12.73	0.000	.148365 .2025226
_cons	9.24379	.013717	673.89	0.000	9.216842 9.270738
N	525				
Adjusted R-squared	0.9559				

**Table 13:** Regression-based decomposition of inequality on log yearly wage in 2003

Decomp.	100*s_f	S_f	100*m_f/m	CV_f	CV_f/CV(total)
Residual	4.3137	0.0022	−0.0000	−1.24e+14	−2.48e+15
Female	13.9683	0.0070	−1.6232	−1.2692	−25.3179
White collars	22.6114	0.0113	1.8178	1.4695	29.3129
Executives	14.1250	0.0071	0.1782	10.2881	205.2227
Trainees	4.3639	0.0022	−0.1502	−3.7473	−74.7502
Middle managers	14.3957	0.0072	0.2835	6.5247	130.1521
30–50 years old	15.0218	0.0075	2.1265	0.9428	18.8061
Over 50 years old	8.7032	0.0044	0.5965	2.8024	55.9004
Central Italy	−0.0126	−0.0000	0.1862	2.0403	40.6984
Islands	0.1882	0.0001	−0.0304	−3.7269	−74.3422
North Eastern Italy	−0.2966	−0.0001	0.3543	1.7366	34.6405
North Western Italy	2.6180	0.0013	0.6104	1.4063	28.0526
Total	100.0000	0.0501	100.0000	0.0501	1.0000

**Table 14:** Econometric regression on log yearly wage in 2013

Log yearly wage 2013	Coef.	Std. Err.	t	P > |t|	[95% Conf. Interval]
Female	−0.472304	.007747	−60.97	0.000	−0.487 −0.457
White collars	0.6509091	.0083336	78.11	0.000	0.634 0.667
Executives	2.025904	.0427336	47.41	0.000	1.941 2.109
Trainees	0.128903	.0172828	7.46	0.000	0.094 0.162
Middle managers	1.3665	.0229033	59.66	0.000	1.321 1.411
30–50 years old	0.5239184	.0096293	54.41	0.000	0.505 0.542
Over 50 years old	0.6508425	.0120905	53.83	0.000	0.627 0.674
Central Italy	0.1940378	.0122788	15.80	0.000	0.169 0.218
Islands	−0.0202988	.0166329	−1.22	0.223	−0.052 0.012
North Eastern Italy	0.3077266	.0118942	25.87	0.000	0.284 0.331
North Western Italy	0.3243027	.0113114	28.67	0.000	0.302 0.346
_cons	8.863065	.0120032	738.39	0.000	8.839 8.886
N	540				
Adjusted R-Squared	0.9753				

**Table 15:** Regression-based decomposition of inequality on log yearly wage in 2013

Decomp.	100*s_f	S_f	100*m_f/m	CV_f	CV_f/CV(total)
Residual	2.4186	0.0014	−0.0000	−8.36e+13	−1.46e+15
Female	14.0112	0.0080	−2.0897	−1.1677	−20.4340
White collars	24.7073	0.0141	2.2758	1.4112	24.6944
Executives	10.9045	0.0062	0.1621	11.3916	199.3423
Trainees	−1.3606	−0.0008	0.0799	3.9854	69.7410
Middle managers	17.0695	0.0098	0.3972	5.9164	103.5308
30–50 years old	12.9800	0.0074	3.0988	0.8761	15.3314
Over 50 years old	12.2709	0.0070	1.2363	2.1231	37.1524
Central Italy	−0.2967	−0.0002	0.4189	1.9610	34.3149
Islands	0.1232	0.0001	−0.0150	−3.6252	−63.4375
North Eastern Italy	1.7519	0.0010	0.7688	1.7853	31.2416
North Western Italy	5.4202	0.0031	1.0811	1.4621	25.5848
Total	100.0000	0.0571	100.0000	0.0571	1.0000

**Table 16:** Econometric regression on log yearly wage in 2018

Log yearly wage 2018	Coef.	Std. Err.	t	P > |t|	[95% Conf. Interval]
Female	−.4681434	.0081762	−57.26	0.000	−.4842049 –.4520818
White collars	.6842877	.0088642	77.20	0.000	.6668747 .7017006
Executives	2.071677	.0487108	42.53	0.000	1.975988 2.167366
Trainees	.2224362	.0184795	12.04	0.000	.1861347 .2587377
Middle managers	1.421381	.0250223	56.80	0.000	1.372227 1.470536
30–50 years old	.6039673	.010097	59.82	0.000	.5841326 .623802
Over 50 years old	.7642786	.0119016	64.22	0.000	.7408988 .7876584
Central Italy	.1842535	.0130152	14.16	0.000	.158686 .2098209
Islands	−.0306534	.0180946	−1.69	0.091	−.066199 .0048921
North Eastern Italy	.3050324	.0125661	24.27	0.000	.2803474 .3297175
North Western Italy	.3210475	.0120214	26.71	0.000	.2974323 .3446626
_cons	8.770863	.0126168	695.17	0.000	8.746079 8.795648
N	545				
Adjusted R-squared	0.9748				

**Table 17:** Regression-based decomposition of inequality on log yearly wage in 2018

Decomp.	100*s_f	S_f	100*m_f/m	CV_f	CV_f/CV(total)
Residual	2.4683	0.0015	0.0000	5.85e+13	9.70e+14
Female	12.1131	0.0073	−2.0792	−1.1659	−19.3261
White collars	25.3352	0.0153	2.3283	1.4423	23.9075
Executives	8.9335	0.0054	0.1431	12.2826	203.5905
Trainees	−1.8550	−0.0011	0.1313	4.0945	67.8682
Middle managers	15.8846	0.0096	0.3880	6.1168	101.3897
30–50 years old	12.7836	0.0077	3.2240	0.9812	16.2638
Over 50 years old	18.1324	0.0109	1.8199	1.8445	30.5742
Central Italy	−0.2786	−0.0002	0.4010	1.9536	32.3812
Islands	0.1603	0.0001	−0.0212	−3.7664	−62.4301
North Eastern Italy	1.4800	0.0009	0.7828	1.7563	29.1114
North Western Italy	4.8425	0.0029	1.0701	1.4644	24.2731
Total	100.0000	0.0603	100.0000	0.0603	0.0603

### Wage loss analysis

**Table 18:** Wage loss events by population sub-groups, Pearson’s *χ*^2^ statistic and *χ*^2^ likelihood ratio

		1983–1984		1993–1994		2003–2004		2013–2014		2017–2018	
Gender	*Pearson*	1.2e+05	Pr = 0.000	1.8e+05	Pr = 0.000	2.8e+06	Pr = 0.000	6.9e+03	Pr = 0.000	6.4e+05	Pr = 0.000
	*LR test*	1.3e+05	Pr = 0.000	2.1e+05	Pr = 0.000	2.9e+06	Pr = 0.000	6.9e+03	Pr = 0.000	6.4e+05	Pr = 0.000
Age class	*Pearson*	5.3e+05	Pr = 0.000	5.6e+05	Pr = 0.000	1.6e+05	Pr = 0.000	5.0e+05	Pr = 0.000	7.1e+05	Pr = 0.000
	*LR test*	5.7e+05	Pr = 0.000	6.3e+05	Pr = 0.000	1.6e+05	Pr = 0.000	4.9e+05	Pr = 0.000	7.2e+05	Pr = 0.000
Geographical area	*Pearson*	5.1e+05	Pr = 0.000	9.8e+05	Pr = 0.000	2.4e+06	Pr = 0.000	1.7e+06	Pr = 0.000	1.7e+05	Pr = 0.000
	*LR test*	5.3e+05	Pr = 0.000	8.1e+05	Pr = 0.000	2.6e+06	Pr = 0.000	1.8e+06	Pr = 0.000	1.7e+05	Pr = 0.000
Job class	*Pearson*	8.5e+05	Pr = 0.000	4.0e+05	Pr = 0.000	5.3e+05	Pr = 0.000	1.6e+06	Pr = 0.000	8.4e+06	Pr = 0.000
	*LR test*	8.9e+05	Pr = 0.000	5.3e+05	Pr = 0.000	6.0e+05	Pr = 0.000	1.6e+06	Pr = 0.000	9.2e+06	Pr = 0.000

## Footnotes

1 In the remainder of the paper, whenever not specified, we use the term inequality always with reference to wage inequality.

2 For instance, the seven class schema distinguishes among professional, administrative and managerial employees with higher grade (I); professional, administrative and managerial employees with lower grade and technicians with higher grade (II); routine nonmanual employees with higher grade (IIIa); routine nonmanual workers with lower grade (IIIb); small employers and self-employed workers (IV); supervisors of manual workers; technicians, lower grade (V); skilled manual workers (VI); semi- and unskilled manual workers (VII) (Goldthorpe and McKnight 2006).

3 INPS Open Data are available online on INPS website.

4 As robustness exercise, available upon request, the empirical analysis has been replicated applying as statistical weight the number of first jobs, confirming our results.

5 Our database collects information on the private sector only, therefore the number of observed jobs differs from the total number of employees as reported by ISTAT (see I.stat database, last retrieved 26 July 2021).

6 One of the outcome of the “hot autumn” season in 1969 – characterised by intense social conflict between trade unions and employers – was a revision of the wage indexation mechanism (*scala mobile*) in 1975 with the introduction of a mechanism aimed at ensuring full wage indexation. The so called *punto unico di contingenza* provided for the payment of a contingency allowance equal for all workers, regardless of the job level. While at the beginning Italian employers seemed to accept this “100% inflation hedging mechanism” (Graziani 1998, p.126) thanks to the possibility of discharging the increasing labour cost on a devaluation of the national currency, with the entry into the EMS and the consequent stabilization of the exchange rate between the lira and the mark, they became great opponents of it.

7 Results are reported in Table 18 in the Appendix.

8 In Acemoglu and Autor (2011) the authors make the example “of software that connects spelling and identifies grammatical errors” (p.82) as machines that substitute routine task. However, machine learning still requires human supervision and direct intervention (Tubaro and Casilli 2019; Casilli 2020).

9 On the one hand, although the labour process in manufacturing might be viewed as standardised, manual workers do perform a large number of cognitive activities related to the control of production and tracking of errors (Pfeiffer 2016; Cetrulo et al. 2020a), which are barely routinised. On the other hand, standardisation is present in all activities, even those that apparently are very far from routinisation, as writing papers, proving theorems or coding software.
